# Comparative effectiveness of physical activity interventions on cognitive functions in children and adolescents with Neurodevelopmental Disorders: a systematic review and network meta-analysis of randomized controlled trials

**DOI:** 10.1186/s12966-024-01702-7

**Published:** 2025-01-13

**Authors:** Ruiyuan Tao, Yijian Yang, Mark Wilson, Jeremy R. Chang, Chang Liu, Cindy H. P. Sit

**Affiliations:** 1 Department of Sports Science and Physical Education, The Chinese University of Hong Kong, Hong Kong SAR, China; 2 Department of Public Health and Sport Sciences, University of Exeter, Exeter, UK; 3 Department of Rehabilitation Sciences, The Hong Kong Polytechnic University, Hong Kong SAR, China; 4 Vanke School of Public Health, Tsinghua University, Beijing, China

**Keywords:** Physical activity, Cognitive functions, Child, Adolescent, Neurodevelopmental disorders, Network meta-analysis

## Abstract

**Background:**

Physical activity (PA) interventions have been shown to yield positive effects on cognitive functions. However, it is unclear which type of PA intervention is the most effective in children and adolescents with Neurodevelopmental Disorders (NDDs). This study aimed to compare the effectiveness of different types of PA interventions on cognitive functions in children and adolescents with NDDs, with additional analyses examining intervention effects across specific NDD types including attention-deficit/hyperactivity disorder (ADHD) and autism spectrum disorder (ASD).

**Methods:**

In this systematic review and network meta-analysis, seven databases (Web of Science, PubMed, Medline, APA PsycINFO, Embase, CINAHL, and SPORTDiscus) for randomized controlled trials from database inception to September 2023 were searched. Randomized controlled trials comparing the effectiveness of PA intervention with any non-pharmacological treatment or control group on cognitive functions in children and adolescents diagnosed with NDDs aged 5–17 years were included. Frequentist network meta-analyses were performed based on standardized mean differences (SMD) using random effects models to examine post-intervention differences in cognitive functions, including attention, memory, and executive functions. Intervention dropout was assessed as a measure of treatment acceptability.

**Results:**

Thirty-one randomized controlled trials (*n* = 1,403, mean age 10.0 ± 1.9 years) with 66 arms were included in the network. Mind-body exercise (MBE; SMD = 1.91 for attention; 0.92 for executive functions), exergaming (SMD = 1.58 for attention; 0.97 for memory; 0.94 for executive functions), and multi-component physical activity (MPA; SMD = 0.79 for executive functions) were associated with moderate to substantial cognitive improvements compared with usual care, whereas the effectiveness of aerobic exercise (AE) was non-significant. Exergaming (SMD = 0.78, 95%CI 0.12 to 1.45) and MPA (SMD = 0.64, 95%CI 0.11 to 1.18) were more effective than AE for executive functions. When analyzing specific NDD types, exergaming lost its superiority over usual care for attention and memory in ADHD, nor for executive functions in ASD. Instead, MPA demonstrated significant benefits across these domains and populations. The certainty of evidence for these comparisons was very low to low. No significant differences in acceptability were observed among MBE, exergaming, and MPA.

**Conclusions:**

The findings in this study suggest that MBE, exergaming, and MPA were effective interventions for improving domain-specific cognitive functions in children and adolescents with NDDs. AE demonstrated non-significant effectiveness for all outcomes. MBE emerges as particularly advantageous for attention. MPA yielded consistent improvements in memory and executive functions across NDD types. Further high-quality randomized controlled trials of direct comparisons are needed to confirm and expand on the findings from this NMA.

**Trial registration:**

PROSPERO CRD42023409606.

**Supplementary Information:**

The online version contains supplementary material available at 10.1186/s12966-024-01702-7.

## Background

Neurodevelopmental Disorders (NDDs) encompass a broad spectrum of early-onset conditions characterized by cognitive and developmental impairments that can persist throughout a lifetime, including attention-deficit/hyperactivity disorder (ADHD), autism spectrum disorder (ASD), intellectual disability (ID), specific learning disorders (SLD), communication disorders, and motor disorders [1]. An estimated 15% of children worldwide are affected by NDDs [2, 3]. Despite the substantial heterogeneity observed in NDDs, their shared molecular pathways underlying clinical signs [4], along with their prominent early-onset neurocognitive deficits and significant symptomatic overlap [5], provide the rationale for grouping these disorders. Supporting this perspective, a recent meta-analysis has indicated that children and adolescents with different types of NDDs may be grouped together when delivering physical activity (PA) interventions [6]. Furthermore, the frequent occurrence of comorbidity among multiple NDDs [1, 2] suggests that exclusively targeting a single disorder may influence the intervention effectiveness when other co-occurring disorders are present [5].

Cognitive deficits in children and adolescents with NDDs are thought to be associated with academic underachievement [7, 8] and social isolation that in turn have a detrimental impact on their quality of life [9, 10], imposing heavy burdens on the individuals themselves, their caregivers, and society. Thereby, early remedial interventions for cognitive functions are of high priority during childhood and adolescence, as these are sensitive periods for shaping cognitive and behavioral developmental processes [11, 12]. Cognitive function domains can be comprehended through diverse conceptualizations, including categorization based on the overarching processes they entail, such as attention, memory, or executive functions [13]. Understanding and implementing effective interventions for the development of attention, memory, and executive functions is crucial, as deficits in these processes often hinder both academic and professional development [14]. Achieving improvements in these cognitive processes can be accomplished through pharmacotherapy [15] and non-pharmacological interventions [16, 17] for individuals with developmental disabilities. The uncertain long-term balance between benefits and side effects associated with medication and low adherence (e.g., 46% in youths with ADHD) [18–20], means that non-pharmacological cognitive enhancers emerge as an attractive option, due to their relatively low risks to well-being, cost-effectiveness, and accessibility to the general population.

PA interventions have gained prominence as a promising strategy to enhance cognitive functions, showing relatively larger effect sizes on specific cognitive domains compared to certain non-pharmacological interventions [21, 22]. For instance, PA interventions demonstrated large effectiveness (effect size = 0.910) in enhancing executive functions in children with ADHD, whereas other non-pharmacological treatments (e.g., cognitive training, Neurofeedback) showed relatively lower effects (ranging from 0.216 to 0.724) [22]. Modifications in brain structure and function may explain the cognition-enhancing effects of PA participation [23], such as the release of monoamine neurotransmitters induced by exercise [24]. Meanwhile, cognitive components (e.g., attention and concentration) can be described as intrinsic factors influencing engagement in PA [25]. Given children with NDDs presented significantly lower adherence to the 24-h movement guidelines [26] compared to peers with typical development [27], it is crucial to implement PA interventions, highlighting the mutual benefits offered in terms of both cognitive enhancement and PA engagement.

In the context of NDDs, prior systematic reviews and pairwise meta-analyses have synthesized that PA interventions were supported as an effective approach for cognitive functions [28–31]. However, the aggregation of various PA interventions when calculating effect size in pairwise meta-analyses restricts their ability to make comparisons across different interventions and consider indirect effects [32]. Consequently, these analyses provide limited insights into the overall effectiveness hierarchy of PA interventions, leaving the question of which specific type of PA intervention is the most effective unanswered. This question is of note considering previous evidence highlighting variations in cognitive benefits associated with different exercise modalities [33, 34]. For example, executive function improvements were only observed in PA interventions with cognitive engagement but not in pure aerobic exercise in children with ASD [35]. As such, this study utilized network meta-analysis (NMA) to systematically investigate and rank different types of PA interventions based on their effectiveness in improving cognitive functions in children and adolescents with NDDs. NMA is a better technique that enhances the precision of pairwise meta-analysis by simultaneously comparing multiple treatments within a single analysis, and incorporating both direct and indirect evidence. This allows for the estimation of comparative effects not directly investigated in randomized controlled trials and the ranking of multiple competing interventions [36]. NMA findings in this study can inform clinicians, educators, and caregivers in selecting the appropriate PA intervention for cognitive functions in children and adolescents with NDDs.

## Methods

This systematic review with NMA was conducted according to the Preferred Reporting Items for Systematic Reviews and Meta-Analyses (PRISMA) statement and PRISMA extension for NMA [37] (Supplementary 1). The protocol was registered in PROSPERO (CRD42023409606).

### Search Strategy and selection criteria

Seven electronic databases (CINAHL, Embase, Medline, PsycINFO, PubMed, SPORTDiscus, and Web of Science) were systematically searched from their inception until 4 September 2023. To avoid missing potentially eligible literature, the reference lists of previous systematic reviews [28, 29, 31, 38–42] and included studies were further examined as complementary sources. The search was restricted to articles written in English. The detailed search strategy is presented in Supplementary 2. The screening and selection processes were conducted independently by two reviewers (RT and CL). Any discrepancies were resolved by consensus. Following the population, intervention, comparison, and outcomes (PICO) framework, studies were eligible if they (1) were randomized controlled trials to assess the effectiveness of any PA intervention in children and adolescents aged 5–17 years; (2) recruited participants diagnosed with NDDs based on clinical assessments or criteria outlined in the Diagnostic and Statistical Manual of Mental Disorders 4th editions or other standardized diagnostic criteria (e.g., Autism Diagnostic Observation Schedule 2nd Edition, International Classification of Diseases 10th editions); (3) received PA intervention and had a comparison arm receiving either any PA intervention or non-pharmacological treatment, or usual care control; and (4) reported at least one cognitive function outcome (i.e., attention, memory, or executive functions). Studies were excluded if they (1) implemented acute PA intervention (i.e., a single bout of exercise); (2) combined PA with other non-pharmacological or pharmacological treatments; (3) recruited children with typical development as control; or (4) were reviews, observational studies, case reports, letters to the editor, or conference abstracts.

We identified four categories of PA interventions based on the definitions described in the Physical Activity Guidelines for Americans [43] and previous systematic reviews [39, 44, 45] or the treatment names assigned by the study authors. The definitions of PA interventions and comparators are presented in Table 1.

**Table 1:** Treatment nodes included in the network meta-analysis

Nodes	Definition
***PA Intervention***	
Aerobic Exercise (AE)	Activities aiming to enhance cardiovascular fitness, including brisk walking, running, and cycling
Exergaming	Exergaming is a form of entertainment that combines physical activity and video gaming, involving body movements during console play [46], such as Nintendo Wii and Xbox Kinect
Mind-body Exercise (MBE)	Exercise that is characterized by controlled physical movements, full body stretching, breathing technique, and a meditation component, such as Yoga, Qigong, Tai Chi, and body-oriented or movement-based mind–body intervention [47, 48]
Multi-component Physical Activity (MPA)	A combination of more than one type of physical activity, such as racket sports which combines AE and coordinative physical activities
***Comparator***	
Relaxation Techniques (RT)	Techniques aim to produce the body’s natural relaxation response, characterized by slower breathing, lower blood pressure, and a feeling of increased well-being, such as guided imagery, progressive muscle relaxation, and breathing techniques [49]
Neurofeedback (NF)	A non-invasive electroencephalograph (brainwave) biofeedback that increases brainwave activity for the purpose of encouraging brain to change and adapt neuroplasticity [50]
Usual Care (UC)	Including no treatment, waiting-list control, or interventions that could not be classified into the other treatment nodes (e.g., educational activities)

### Outcomes

The primary outcomes were cognitive functions, including attention, memory, and executive functions. Attention indicates the ability to focus on relevant information while disregarding nonrelevant stimuli and maintain sustained attention over time [13]. Memory, the most complex cognitive domain possessing multifaceted subdomains (e.g., working memory, episodic memory, semantic memory), involves the sequential stages of encoding, storage, and retrieval [13]. Executive functions refer to cognitive processes such as reasoning, problem-solving, planning, strategizing, decision-making, inhibiting irrelevant information, and task-switching [13]. The secondary outcome was treatment acceptability, defined as the proportion of participants who completed their assigned treatment (PA intervention or comparison).

### Data extraction

Relevant information was extracted standardly by two independent reviewers (RT and CL), including bibliographic data (author, publication year, country/region), participant characteristics (diagnosis, sample size, age, gender, medication use), intervention components (category, frequency, duration, length, and intensity), and primary outcome measurements at immediate post-intervention. When studies used two or multiple measures to assess the same cognitive function domain, the most commonly used task was included [51]. If studies provided multiple raw scores for a single task, the outcome with higher quality was selected (e.g., interference is assessed as high quality in Stroop task). This score of quality was developed by Op den Kelder et al. [52] and adapted by Johnson et al. [53], indicating the specificity of measurements corresponding to the cognitive domain. When means and standard deviations changes from baseline were incompletely reported, we calculated them using the formula provided in the Cochrane Handbook [54]. If data were missing, the corresponding authors were directly contacted through emails to request additional information.

The Cochrane risk-of-bias tool for randomized trials [55] was used to assess the methodological quality of included randomized controlled trials. The certainty of evidence for attention, memory, and executive functions was appraised within the Confidence in Network Meta-Analysis (CINeMA) framework [56, 57].

### Data analysis

Random effects pairwise meta-analyses with the Hartung-Knapp-Sidik-Jonkman method [58] were first conducted to summarize all direct comparisons within the included studies. The effect size was calculated as standardized mean differences (SMD) for continuous data (effectiveness) and odds ratios (OR) for dichotomous data (acceptability) with 95% confidence intervals (95%CIs). SMD of 0.2, 0.5, and 0.8 are interpreted as small, moderate, and large, respectively [59]. In our investigation, we integrated data from four categories of PA interventions to synthesize the collective effects on attention, memory, and executive functions, as well as their acceptability in comparison to usual care. When encountering multi-arm studies, the sample size of the shared group was split into subgroups of equal size, one for each treatment [60].

Random-effects network meta-analyses were performed using the frequentist framework in Stata 17 and R 4.0.4 to compare the relative effectiveness and treatment acceptability through direct and indirect comparisons. To evaluate the assumptions underlying the NMA, transitivity was judged by visualizing the relative distribution of potential effect modifiers (e.g., sample size, age, gender, intervention length, and dose) across interventions. Transitivity implies that the included studies possess similarity in terms of potential effect modifiers. We scrutinized the consistency of the results. Global inconsistency, reflecting overall inconsistencies across treatment comparisons, was assessed using the design-by-treatment inconsistency model [61]. Local inconsistency, which examines discrepancies within individual nodes or treatment comparisons, was evaluated through the node-splitting method [62]. The heterogeneity among the included studies was determined by calculating the between-study variance (τ^2^) and comparing it with empirical estimates [63, 64]. Treatment rankings were established using the surface under the cumulative ranking curve (SUCRA) which is a precise estimation to provide a hierarchy of the treatments [65].

Additionally, we examined the comparative effectiveness of PA interventions across different NDD types. Specifically, we examined comparative outcomes in attention, memory, and executive functions for ADHD, and executive functions for ASD. Due to the limited number of studies available, DCD (*n* = 2) and SLD (*n* = 1) were not included in this analysis. To assess the robustness of our findings and identify potential sources of heterogeneity, we conducted sensitivity analyses by removing studies with high risk of bias. Meta-regression analyses were performed to investigate the influence of sample size, intervention length, and dose on primary analysis. Subgroup analysis was performed based on NDD category, intervention duration, frequency, length, and intensity. Since undertaking subgroup analysis requires a minimum of ten studies [66], we restricted this analysis to memory and executive functions. Small-study effects were evaluated by the comparison-adjusted funnel plot and the Egger test [67].

## Results

**Fig. 1:**
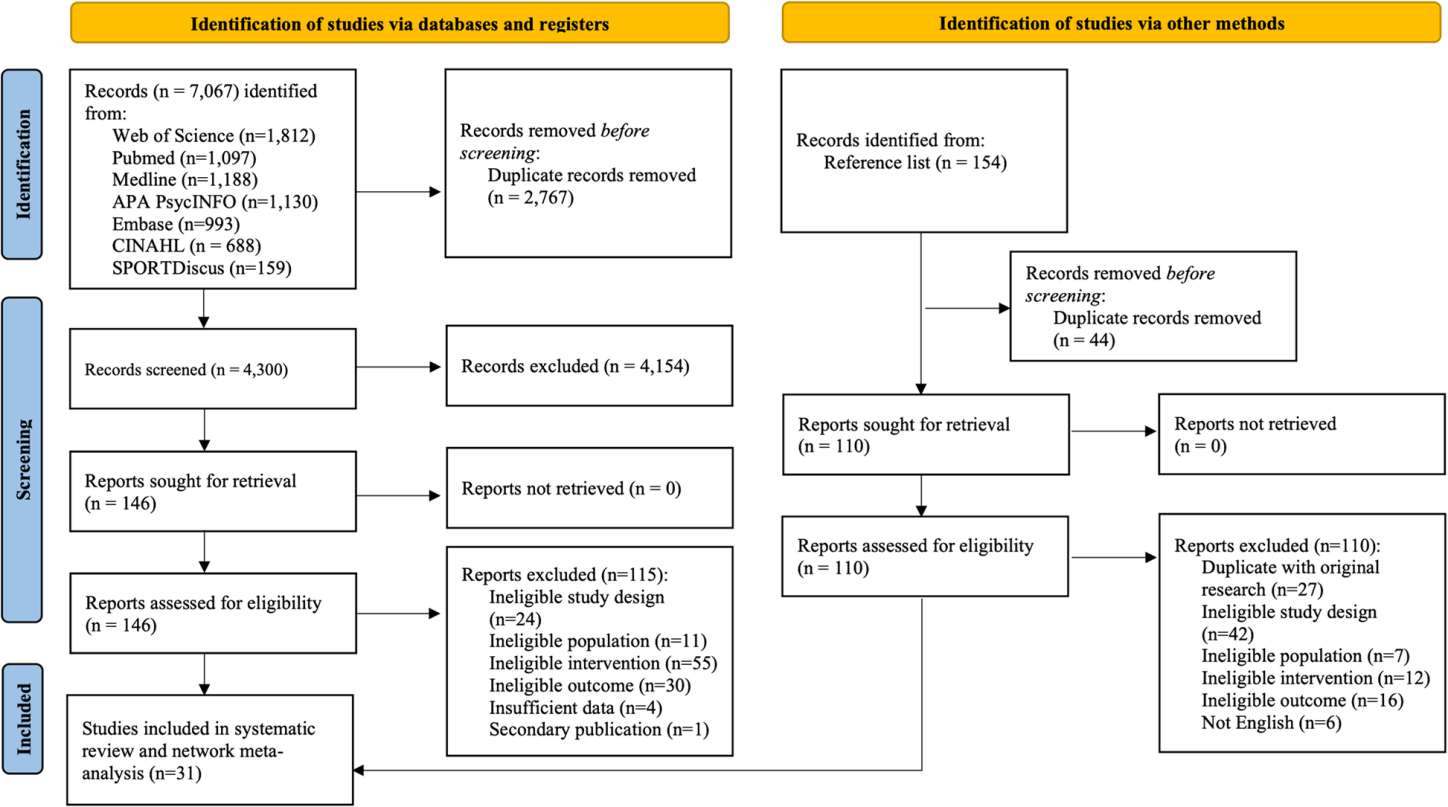
PRISMA study selection and flow chart

Overall, 7,067 records were initially identified from electronic databases. After removing duplicates, 4,300 records were screened for titles and abstracts, and 146 full-text articles were retrieved for eligibility. An additional 154 records identified from reference lists of relevant systematic reviews were also screened for eligibility (Fig. 1). Eventually, a total of 31 studies [35, 68–97] involving 1,403 children and adolescents with NDDs were included in this review.

### Study characteristics

Table 2 summarizes detailed information on each included study. The mean age of participants was 10.0 ± 1.9 years with 83.2% being boys. The majority of included studies focused on ADHD (*n* = 18). MPA (*n* = 19) was the most frequently utilized PA modality. All studies reported immediate post-intervention assessments (attention: *n* = 10; memory: *n* = 11; executive functions: n = 26). Supplementary 3 shows the general characteristics of the included studies.

**Table 2:** Characteristics of included studies

Study (year; country/region)	Participants; Diagnostic Methods	Sample Size	Age (years)	Gender (% male)	Medication	Treatment	Node	Length	Duration per session	Frequency	Intensity	Cognitive Function Outcomes
Ahmed & Mohamed, 2011 [68] Egypt	ADHD; Clinical	42	11.0–16.0	64.3	NI	Running	AE	10 weeks	40–50 min	3 sessions/week	Moderate	Attention: MCRS
		42			NI	No treatment control	UC					
Benzing & Schmidt, 2019 [69] Switzerland	ADHD; ICD-10	28	10.5 ± 1.3	86.4	Y	Xbox Kinect: workout games	Exergaming	8 weeks	30 min	3 sessions/week	MVPA	Memory: CSBT EF: Simon Task, Flanker Task
		23	10.4 ± 1.4	81.8		Waiting-list control	UC					
Borgi et al., 2016 [70] Italy	ASD; DSM-IV	15	9.2 ± 1.8	100.0	NI	Equine-assisted therapy	MPA	6 months	60–70 min	1 session/week	NI	EF: TOL
		13	8.0 ± 1.5	100.0		Waiting-list control	UC					
Bustamante et al., 2016 [71] USA	ADHD; DSM-IV	19	9.4 ± 2.2	69.0	Y	Aerobic activity	AE	10 weeks	60 min	5 sessions/week	MVPA	Memory: AWMA-S EF: SST, BRIEF
		16	8.7 ± 2.0	68.0		Sedentary arts activity	UC					
Chan et al., 2013 [72] Hong Kong SAR	ASD; DSM-IV	23	11.3 ± 3.9	95.0	N	Nei Yang Gong	MBE	4 weeks	18 min	3–6 sessions/week	Moderate	EF: TOL, CCTT-T2, FPT
		23	12.4 ± 3.3	85.0		Progressive muscle relaxation	RT					
Chang et al., 2022 [73] Taiwan	ADHD; DSM-V	16	8.3 ± 1.3	81.3	Y	Table tennis	MPA	12 weeks	60 min	3 sessions/week	NI	EF: Stroop Task, WCST
		16	8.4 ± 1.2	81.3		Nintendo Wii fit training	Exergaming	12 weeks	60 min	3 sessions/week	NI	
		16	8.4 ± 1.3	81.3		No treatment control	UC					
Choi et al., 2015 [74] Korea	ADHD; DSM-IV	17	15.8 ± 1.7	100.0	Y	Running, rope-jumping, basketball	MPA	6 weeks	90 min	3 sessions/week	Moderate	EF: WCST
		18	16.0 ± 1.2	100.0		Educational sessions	UC					
Damanpak & Sabzi., 2022 [75] Iran	DCD; DCDQ	15	10.8 ± 0.4	100.0	NI	Sensory-motor games	MPA	8 weeks	45–60 min	3 sessions/week	NI	EF: CEFS
		15	10.6 ± 0.5	100.0		No treatment control	UC					
Emami Kashfi et al., 2019 [76] Iran	SLD; Psychologist	15	8.7 ± 0.6	NI	NI	Perceptual-motor activities	MPA	8 weeks	55 min	3 sessions/week	NI	Attention: CPT Memory: N-Back test EF: TOL
		15	8.5 ± 0.7	NI		Regular educational services	UC					
Geladé et al., 2017 [77] Netherlands	ADHD; DSM-IV	37	9.8 ± 2.0	75.7	NI	Repetitive interval training	AE	28 sessions	45 min	3 sessions/week	MVPA	Attention: AOT Memory: VSWM EF: SST
		39	10.0 ± 1.9	76.9		Neurofeedback	NF					
Greco & De Ronzi, 2020 [78] Italy	ASD; ADOS-2	14	9.1 ± 1.0	85.7	NI	Karate	MBE	12 weeks	45 min	2 seesions/week	NI	EF: BRIEF
		14	9.4 ± 1.0	85.7		Waiting-list control	UC					
Hashmi et al., 2022 [79] Iran	DCD; DSM-V	25	9.4 ± 2.1	100.0	NI	Nintendo Wii fit training	Exergaming	8 weeks	30 min	3 sessions/week	NI	Attention: CAS-attention Memory: TVPA-R EF: CAS-planning
		25	9.7 ± 2.4	100.0		Usual care	UC					
Hattabi et al., 2019 [80] Tunisia	ADHD; DSM-IV	20	10.0 ± 1.3	85.0	NI	Aquatic activities	MPA	12 weeks	90 min	3 sessions/week	Moderate	Memory: ROCF EF: Stroop, JHT
		20	9.8 ± 1.3	90.0		No treatment control	UC					
Ji & Yang, 2022 [81] China	ASD; ICD-10	49	13.1 ± 3.0	51.5	NI	Football	MPA	6 weeks	60 min	3 sessions/week	NI	Attention: MOT
		50	12.8 ± 2.7	54.6		Psychological counseling	UC					
Ji et al., 2023 [82] Republic of Korea	ADHD; Clinical	21	9.0 ± 1.5	88.0	Y	Alchemist’s Treasure	Exergaming	4 weeks	50 min	3 sessions/week	MVPA	Attention: FAIR
		19	8.9 ± 1.6	86.0	Y	Stationary bike exercise	AE	4 weeks	50 min	3 sessions/week		EF: GNG
Kadri et al., 2019 [83] Tunisia	ADHD; Psychologist	20	14.5 ± 3.5	90.0	N	Taekwondo	MBE	1.5 years	50 min	2 seesions/week	NI	Attention: Ruff 2 & 7 EF: Stroop Task
		20	14.2 ± 3.0	90.0	N	Usual PE class	UC					
Lee et al., 2017 [84] Republic of Korea	ADHD; DSM-IV	6	8.8 ± 1.0	100.0	N	Ball and Rope-jumping	MPA	12 weeks	60 min	3 sessions/week	MVPA	EF: Stroop
		6	8.8 ± 1.0	100.0	N	No treatment control	UC					
Liang et al., 2022 [85] China	ADHD; DSM-V	40	8.4 ± 1.4	30.8	N	AE and ball games	MPA	12 weeks	60 min	3 sessions/week	MVPA	EF: TOL, Flanker Task, TMT
		40	8.3 ± 1.3	32.8	N	Waiting-list control	UC					
Ludyga et al., 2022 [86] Switzerland	ADHD; DSM-V	31	10.8 ± 1.2	62.1	NI	Judo	MBE	12 weeks	60 min	2 sessions/week	Moderate	Memory: CDT
		32	10.0 ± 1.2	79.3		Waiting-list control	UC					
Memarmoghaddam et al., 2016 [87] Iran	ADHD; Psychiatrist	20	8.3 ± 1.3	100.0	N	Running and ball games	MPA	8 weeks	90 min	3 sessions/week	MVPA	EF: Stroop Task, GNG
		20	8.3 ± 1.3	100.0		No treatment control	UC					
Nejati & Derakhshan, 2021 [88] Iran	ADHD; DSM-V	15	9.4 ± 1.4	86.7	NI	Goal-directed exercise	MPA	4–5 weeks	40–50 min	3 sessions/week	NI	Memory: One-Back Task EF: GNG, WCST
		15	9.7 ± 2.4	86.7		Running	AE	4–5 weeks	40–50 min	3 sessions/week		
Pan et al., 2016 [89] Taiwan	ADHD; DSM-IV	16	8.9 ± 1.5	100.0	Y	Racket sport	MPA	12 weeks	70 min	2 sessions/week	NI	EF: Stroop Task
		16	8.9 ± 1.6	100.0		Waiting-list control	UC					
Pan et al., 2017 [90] Taiwan	ASD; DSM-IV	11	9.7 ± 1.6	100.0	Y	Racket sport	MPA	12 weeks	70 min	2 sessions/week	NI	EF: WCST
		11	8.5 ± 1.8	100.0		Waiting-list control	UC					
Phung & Goldberg, 2019 [91] USA	ASD; ADOS-2	14	9.1 ± 1.1	100.0	NI	Martial Arts	MBE	13 weeks	45 min	2 seesions/week	LMPA	EF: Hearts & Flowers test
		20	9.5 ± 1.1	70.0		Waiting-list control	UC					
Rafiei Milajerdi et al., 2021 [92] Iran	ASD; ADOS-2	20	8.2 ± 1.5	95.0	Y	SPARK	MPA	8 weeks	35 min	3 sessions/week	LMPA	EF: WCST
		20	8.0 ± 1.6	95.0		Exergaming	Exergaming	8 weeks	35 min	3 sessions/week	LMPA	
		20	8.5 ± 1.4	95.0		No treatment control	UC					
Rezaei et al., 2018 [93] Iran	ADHD; DSM-V	7	7.0–11.0	NI	NI	Yoga	MBE	8 weeks	45 min	3 sessions/week	NI	Attention: CPT Memory: WISC-LNST
		7		NI		Neurofeedback	NF					
		7		NI		No treatment control	UC					
Sani et al., 2022 [94] Iran	ADHD; DSM-V	25	7.5 ± 1.3	80.0	NI	Perceptual-motor exercises	MPA	20 sessions	40–45 min	3 sessions/week	NI	Attention: CPT
		25	7.8 ± 1.3	65.0	NI	Neurofeedback	NF					
Silva et al., 2020 [95] Brazil	ADHD; DSM-IV	18	12.0 ± 2.0	80.0	Y	Aquatic activities	MPA	8 weeks	45 min	2 seesions/week	NI	Attention: CAT EF: The Test of Trails
		15	12.0 ± 1.0	60.0	Y	No treatment control	UC					
Tse et al., 2019 [96] Hong Kong SAR	ASD; DSM-V	25	10.1 ± 1.2	73.7	Y	Basketball	MPA	12 weeks	45 min	2 sessions/week	Moderate	Memory: CBTT, DSFBT EF: GNG
		25	9.8 ± 1.2	85.7	Y	No treatment control	UC					
Tse et al., 2021 [35] Hong Kong SAR	ASD; DSM-V	22	9.6 ± 1.6	80.0	Y	Stationary cycling	AE	2 weeks	60 min	5 sessions/week	LMPA	Memory: CBTT, DSFBT EF: TOL, GNG, SCWT
		22	10.2 ± 1.7	86.4	Y	Mobile cycling	MPA	2 weeks	60 min	5 sessions/week	LMPA	
		22	9.9 ± 1.3	75.0	Y	No treatment control	UC					
Tse et al., 2023 [97] Hong Kong SAR	ASD;	25	9.6 ± 1.4	87.0	Y	Mobile cycling	MPA	2 weeks	60 min	5 sessions/week	LMPA	EF: SCWT, GNG
	SRS-2	25	10.2 ± 1.4	68.4	Y	Stationary cycling	AE	2 weeks	60 min	5 sessions/week	LMPA	

*ADHD* attention-deficit/hyperactivity disorder, *ASD* autism spectrum disorder, *DCD* developmental coordination disorder, S*LD* specific learning disorder; *ADOS-2* Autism Diagnostic Observation Schedule-2nd Edition, *DCDQ* The Developmental Coordination Disorder Questionnaire, *DSM-IV/V* Diagnostic and Statistical Manual of Mental Disorders-4th/5th editions, *ICD-10* International Classification of Diseases, 10th edition, *SNAP-IV* Swanson, Nolan, and Pelham-4th edition, *SRS-2* Social Responsiveness Scale-2nd Edition; *Y* yes, *N* no, *NI* no information; *LMPA* light-to-moderate physical activity, *MVPA* moderate-to-vigorous physical activity; *AE* aerobic exercise, *MBE* mind–body exercise, *MPA* multicomponent physical activity, *RT* relaxation techniques, *NF* neurofeedback, *UC* usual care; *EF* executive functions; *AOT* Auditory Oddball Task, *AWMA-S* Automated Working Memory Assessment System—Short version, *CAS* Cognitive Assessment System, *CAT* Cancellation Attention Test, *CBTT* Corsi block tapping task, *CCTT-T2* Children’s Color Trails Test-Second Trial, *CDT* Change Detection Task, *CEFS* Coolidge Executive Functioning Scale, *CPT* Continuous Performance Test, *CSBT* Color Span Backward Task, *DSFBT* Digit Span Forward and Backward Test, *FAIR* Frankfurter Aufmerksamkeits-Inventar, *FPT* Five Point Test, *GNG* Go/No-Go Task, *JHT* Junior Hayling Test, *LNST* Letter-Number Sequencing Test, *MCRS* Modified Conner’s Rating Scale, *MOT* Multiple Object Tracking Paradigm, *NEPSY-II* Developmental Neuropsychological Assessment, *ROCF* Rey Complex Figure, SCWT Stroop Color and Word Test, *SPARK* Sports, Play and Active Recreation for Kids, *SST* Stop-Signal Task, *TMT* Trail Making Test, *TOL* Tower of London, *TVPA-R* Test of Visual-Perceptual Skills-Revised, *VSWM* Visual Spatial Working Memory Task, *WCST* Wisconsin Card Sorting Test, *WISC* Wechsler Intelligence Scale for Children

### RoB assessment

The domain level and overall risk of bias assessment are presented in Supplementary 4. Twenty-six included studies were rated with some concerns, while five had high risks of bias. The main concerns for risk of bias were the open-label nature of studies, unblinded assessors, and improper management of missing data.

### Pairwise meta-analyses

The pairwise meta-analyses results are presented in Supplementary 5. Significant moderate to large improvements in attention, memory, and executive functions were observed compared with usual care (SMD = 0.46–1.26). However, there were no significant differences among the interventions compared to usual care for acceptability (OR = 0.88, 96%CI: 0.53 to 1.48).

### NMA assumption

The assumption tests for NMA did not reveal significant concerns regarding the violation of the transitivity assumption when assessing the distribution of potential effect modifiers (Supplementary 6), supporting the assumption of comparability between the treatments. No global or local inconsistency was detected (Supplementary 7), indicating the agreement between direct and indirect evidence. The common heterogeneity τ^2^ ranged from 0.08 to 0.68 indicating moderate heterogeneity within the prediction distributions (Supplementary 8).

### Attention

**Fig. 2:**
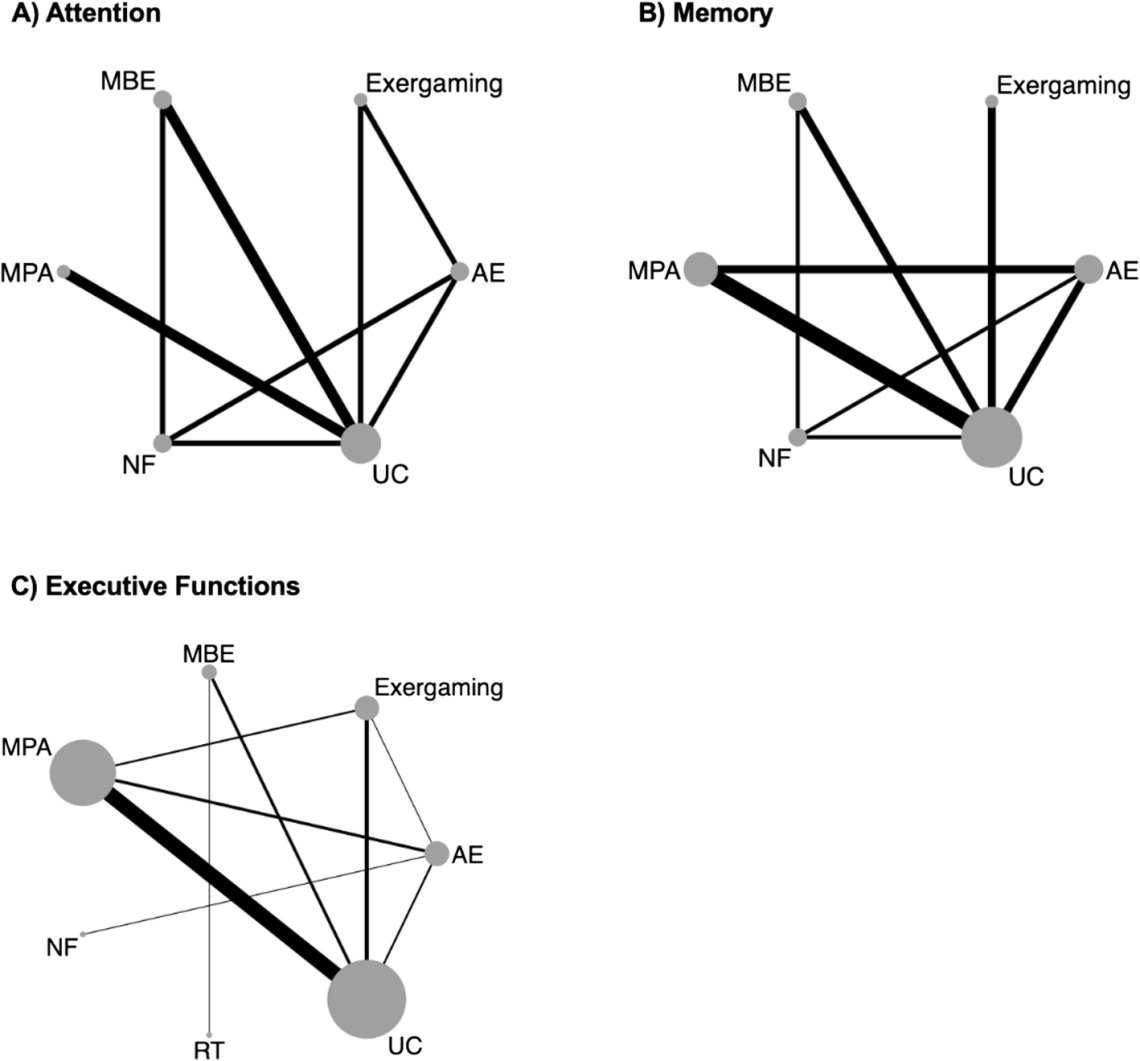
Network plots for attention (**A**), memory (**B**), and executive functions (**C**). Each node represents a treatment. Connecting lines between two nodes represent one or more trials in which the two nodes have been compared directly. Size of each node indicates the number of participants. Thickness of the lines indicates the number of trials that directly compared the treatments it connected. *AE* aerobic exercise, *MBE* mind–body exercise, *MPA* multicomponent physical activity, *RT* relaxation techniques, *NF* neurofeedback, *UC* usual care

Figure 2a presents the network plot for attention, including 10 studies and 441 participants. Table 3 displays the comparative effectiveness of six different treatments. Compared with usual care, large improvements were found for MBE (SMD = 1.91, 95%CI: 0.57 to 3.25) and exergaming (SMD = 1.58, 95%CI: 0.14 to 3.02). The ranking of treatment based on SUCRAs revealed that MBE (SUCRA = 83.4%) was likely the most effective, followed by exergaming (SUCRA = 72.3%)(Supplementary 11). No small-study effect was found using the comparison-adjusted funnel plots and Egger test results (Supplementary 12). When considering effectiveness in specific NDD, available estimates on data from ADHD indicated that MBE (SMD = 2.00, 95%CI: 0.89 to 3.10) and MPA (SMD = 1.87, 95%CI: 0.61 to 3.14) showed more effectiveness over usual care (Supplementary 13).

**Table 3:** League table for attention

MBE					
0.33 (-1.60,2.26)	Exergaming				
0.95 (-0.65,2.56)	0.62 (-1.08,2.32)	MPA			
1.15 (-0.58,2.88)	0.82 (-0.62,2.26)	0.20 (-1.27,1.67)	AE		
1.35 (-0.21,2.92)	1.02 (-0.69,2.74)	0.40 (-0.88,1.69)	0.20 (-1.13,1.54)	NF	
**1.91 (0.57,3.25)**	**1.58 (0.14,3.02)**	0.96 (-0.03,1.95)	0.76 (-0.46,1.99)	0.56 (-0.64,1.75)	UC

*AE* aerobic exercise, *MBE* mind-body exercise, *MPA* multicomponent physical activity, *NF* neurofeedback, *UC* usual care. Results are presented as standardized mean differences (SMD) and 95% confidence intervals. SMD values more than 0.00 favor the column-defining treatment node for the NMA results. Estimates in bold denote significance at *p*<0.05

### Memory

Figure 2b presents the network plot for memory, including 11 studies with 484 participants. Compared with usual care, exergaming yielded significant large beneficial effects (SMD = 0.97, 95%CI: 0.10 to 1.84) (Table 4) and had the highest probability of being the most effective intervention (SUCRA = 88.0%) (Supplementary 11). No small-study effect was observed using the comparison-adjusted funnel plots and Egger test results (Supplementary 12). When evaluating effectiveness within specific NDD, only MPA was more effective than usual care (SMD = 0.81, 95%CI: 0.24 to 1.38) and outperformed AE (SMD = 0.74, 95%CI: 0.14 to 1.35) in ADHD (Supplementary 13).

**Table 4:** League table for memory

Exergaming					
0.48 (-0.56,1.53)	MPA				
0.51 (-0.74,1.76)	0.03 (-1.02,1.08)	MBE			
0.70 (-0.60,2.01)	0.22 (-0.84,1.28)	0.19 (-0.95,1.34)	NF		
0.96 (-0.14,2.07)	0.48 (-0.24,1.21)	0.45 (-0.62,1.53)	0.26 (-0.67,1.19)	AE	
**0.97 (0.10,1.84)**	0.49 (-0.09,1.07)	0.46 (-0.44,1.36)	0.27 (-0.71,1.25)	0.01 (-0.68,0.69)	UC

*AE* aerobic exercise, *MBE* mind-body exercise, *MPA* multicomponent physical activity, *NF* neurofeedback, *UC* usual care. Results are presented as standardized mean differences (SMD) and 95% confidence intervals. SMD values more than 0.00 favor the column-defining treatment node for the NMA results. Estimates in bold denote significance at *p*<0.05

### Executive functions

Figure 2c presents the network plot for executive functions, including 26 studies with 1,003 participants. Exergaming, MBE, and MPA produced large positive effects (SMDs = 0.79–0.94) compared with usual care. Furthermore, exergaming (SMD = 0.78, 95%CI: 0.12 to 1.45) and MPA (SMD = 0.64, 95%CI: 0.11 to 1.18) were significantly superior to AE (Table 5). The SUCRAs revealed that exergaming (SUCRA = 80.6%) was the treatment most likely to perform best, followed by MBE (SUCRA = 78.1%), and MPA (SUCRA = 70.2%) (Supplementary 11). AE did not demonstrate significant effects on executive functions compared to usual care (SMD = 0.15, 95%CI: -0.39 to 0.70). The comparison-adjusted funnel plot and Egger test revealed a certain degree of asymmetry for executive functions (Supplementary 12), suggesting the presence of potential publication bias in this analysis. When analyzing specific NDD types, MBE, MPA, and exergaming were more effective than usual care (SMDs = 0.57–1.82) and outperformed AE (SMDs = 0.66–1.91) in ADHD. In ASD, however, only MPA showed significant effectiveness over usual care (SMD = 0.67) (Supplementary 13).

**Table 5:** League table for executive functions

Exergaming						
0.02 (-0.85,0.89)	MBE					
0.14 (-0.40,0.69)	0.12 (-0.64,0.89)	MPA				
0.55 (-0.89,2.00)	0.53 (-0.62,1.69)	0.41 (-0.98,1.80)	RT			
0.67 (-0.59,1.93)	0.65 (-0.74,2.04)	0.53 (-0.67,1.72)	0.12 (-1.69,1.93)	NF		
**0.78 (0.12,1.45)**	0.76 (-0.13,1.65)	**0.64 (0.11,1.18)**	0.23 (-1.23,1.69)	0.11 (-0.96,1.19)	AE	
**0.94 (0.42,1.45)**	**0.92 (0.21,1.62)**	**0.79 (0.48,1.11)**	0.38 (-0.97,1.74)	0.27 (-0.94,1.47)	0.15 (-0.39,0.70)	UC

*AE* aerobic exercise, *MBE* mind–body exercise, *MPA* multicomponent physical activity, *RT* relaxation techniques, *NF* neurofeedback, *UC* usual care. Results are presented as standardized mean differences (SMD) and 95% confidence intervals. SMD values more than 0.00 favor the column-defining treatment node for the NMA results. Estimates in bold denote significance at *p* < 0.05

### Acceptability

The NMA for treatment acceptability included 31 studies with n = 1,403 participants (Supplementary 9). Apart from AE (OR = 0.38, 95% CI: 0.15 to 0.97), which was slightly less accepted than usual care, no differences in dropout rates were observed among other PA interventions (Supplementary 10).

### Additional analyses

The sensitivity analyses with removing studies with high risk of bias were generally consistent with the original results (Supplementary 14). Meta-regressions did not demonstrate the significant impacts of the sample size, intervention length, or total dose on cognitive parameters (Supplementary 15). Subgroup analyses showed that significant improvements in memory were found in ADHD, DCD, and SLD, but not for ASD (Hedges’ g = -0.06, 95%CI: -0.52 to 0.40, I^2^ = 25.49%). Additionally, PA interventions had significant effects on executive functions when the frequency was no more than three sessions per week at moderate-to-vigorous intensity (Supplementary 14). The certainty of evidence regarding the effectiveness of PA interventions in enhancing attention, memory, and executive functions was very low to low (Supplementary 16). The evidence was downgraded primarily due to concerns about within-study bias, imprecision, and heterogeneity.

## Discussion

This NMA summarized the available evidence and identified that MBE, exergaming, and MPA were substantiated to yield advantageous effects on specific cognitive functions. We noted no significant differences in acceptability among these PA interventions, suggesting they are comparably accepted to usual care. However, AE did not significantly differ from usual care across the three cognitive domains and was less accepted than usual care. The quality of evidence based on the CINeMA framework demonstrated very low to low quality, underscoring the necessity for cautious interpretations of our findings.

The NMA results indicate that MBE was the most effective PA intervention for improving attention. It may be attributed that MBE emphasizes the coordination between breathing, awareness of bodily sensation, and movement performance [98] by combining controlled physical movements and meditation components that foster internal concentration and body awareness [99]. MBE necessitates energy expenditure while simultaneously demanding a high level of concentration. This mindfulness-based intervention entails prolonged engagement in attentional control skills, such as sustained attention and attentional switching, which generally improves attentional control capabilities [100]. Additionally, for ADHD, which is characterized by persistent inattention and/or hyperactivity-impulsivity [1], the results consistently showed MBE as the most effective intervention for improving attention.

When taking all NDDs into consideration, we found that exergaming appeared as the most effective PA intervention for improving memory and executive functions. The benefits of exergaming may be attributed to the fact that cognitive improvements resulting from PA may partially depend on the enjoyable emotions derived from the activities [101, 102]. Unlike repetitive movements and intense exertion often associated with AE, exergaming offers challenges, feedback, and rewards that make PA a more enjoyable and entertaining endeavor [103]. Moreover, video games have been acknowledged as motivators for promoting active behavior engagement [104]. The combination of enjoyment, motivational gains, and immersion in exergaming may collectively contribute to cognitive benefits through the long-term engagement in PA [102]. From the etiological perspective, alterations in brain circuits of NDDs establish a connection between dopaminergic dysfunction and the expression of cognitive capacities [105], which is evident in conditions such as ADHD [106] and ASD [107]. Therefore, another plausible explanation is that enjoyable exergaming triggers the release of dopamine, which is conjectured to enhance cognitive processes [23, 108]. Further empirical studies are warranted to support these conceptual assumptions within the context of NDDs. However, in specific NDD populations, only MPA demonstrated consistently significant effectiveness across three cognitive functions in children and adolescents with ADHD, as well as in executive functions in ASD. The studies classified as MPA in this NMA primarily involved coordinatively demanding and non-automated sport-related activities that were assumed to activate brain regions associated with higher-order cognitive processes [109–111].

Our findings on the superior effectiveness of MPA over AE in improving executive functions align with results from RCT that directly compared these interventions head-to-head [88], but differ from prior meta-analyses [28, 31] that reported comparable cognitive benefits for both AE and cognitively engaging exercises. This discrepancy may partially stem from the methodology. First, both reviews [28, 31] did not limit their inclusion criteria to randomized controlled trials. Pre-experimental or quasi-experimental designs could compromise external validity and introduce potential bias (e.g., regression to mean), which may affect the observed effects [112]. Second, the variability stemming from intervention lengths (i.e., acute or chronic) may potentially account for the disparities in these findings. Upon reviewing the studies included by Liang et al. [28] and Sung et al. [31], the significant effects of AE on cognitive improvements were observed following episodes of acute treadmill walking, running, or stationary cycling in children with NDDs. It is postulated that the intervention effects might be more readily observable when assessed immediately following exercise [41]. These findings are further supported by the temporal dynamics of concentration changes in neurochemicals, suggesting that alterations in cognitive processes and functions following acute exercise were associated with the corresponding changes in neurochemical levels [113]. High-quality randomized controlled trials are warranted to ascertain the effectiveness of long-term AE in children and adolescents with NDDs. Nevertheless, we echoed the appeal of “Going beyond Simply Moving to Moving with Thought” [33], suggesting that future PA interventions could prioritize cognitive efforts over basic repetitive activities that demand little thought.

In terms of intervention acceptability, while MBE, MPA, and exergaming demonstrated comparable outcomes in this NMA, exergaming exhibited relatively lower rankings. Limited comprehension of instructions may lead to noncompliance or attrition with intervention protocols, potentially compromising the intervention’s effectiveness [109]. Therefore, programmatic modifications may be necessary before implementation. Adaptations of exergaming, such as enhancement of visual comprehensibility, may better support the learning and processing needs of children and adolescents with NDDs. Notably, in this NMA, AE demonstrated the lowest acceptability among the included treatments and exhibited significantly lower acceptance rates compared to usual care. The reduced acceptance of AE may be attributed to the inherently repetitive nature of such activities, which often involve continuous, rhythmic movements, such as running, stationary cycling, or other repetitive patterns. The monotonous character of these standardized, sustained actions may affect children’s engagement and motivation. These findings indicate the consideration of incorporating enjoyable and cognitively challenging elements into PA interventions.

### Limitations

Several limitations should be considered when interpreting our results. First, despite our efforts to categorize various types of PA according to guidelines [43] and previous NMAs [44, 45], some included interventions were classified based on the descriptions provided by the authors, which were not standardized. Second, the majority of the included randomized controlled trials had limited sample sizes, potentially undermining the ability to obtain robust and conclusive evidence owing to the small study effect. Similarly, a few included studies on various types of PA led to thinly connected networks and underpowered estimates for detecting possible differences. Third, given that the certainty of evidence was very low to low, the effect sizes and rankings of the treatments are likely to change as more evidence becomes available. Fourth, the limited information available on baseline IQ or medication usage hampers our ability to explore their impacts on the results. Fifth, included studies mainly focused on the effects of chronic PA interventions at immediate post-intervention and predominantly involved males (79.9%) with ADHD or ASD. Future research is needed to evaluate the long-term effectiveness of PA interventions on gender and other NDDs.

## Conclusions

This is the first NMA to estimate the comparative effectiveness of various PA interventions on cognitive functions in children and adolescents with NDDs. Our results revealed that the effectiveness varies according to the PA types. MBE, exergaming, and MPA have significant domain-specific cognitive benefits. AE demonstrated non-significant effectiveness for all outcomes. MBE emerges as particularly advantageous for attention. MPA yielded consistent improvements in memory and executive functions across NDD types. Our results suggest that there is a need to select appropriate PA interventions to improve specific cognitive function in children and adolescents with NDDs. With the low quality of some findings in this NMA, we recommend that direct head-to-head comparison randomized controlled trials are warranted to confirm the relationships between PA and cognitive functions in this population.

## Supplementary Information

Supplementary Material 1.

## Data Availability

All data generated or analysed during this study are included in this published article and its supplementary files.

## Abbreviations

ADHD: Attention-deficit/hyperactivity disorder
AE: Aerobic exercise
ASD: Autism spectrum disorder
CINeMA: Confidence in network meta-analysis
CIs: Confidence intervals
ID: Intellectual disability
MBE: Mind-body exercise
MPA: Multi-component physical activity
NDDs: Neurodevelopmental disorders
NF: Neurofeedback
NMA: Network meta-analysis
OR: Odds ratios
PA: Physical activity
PICO: Population, intervention, comparison, and outcomes
PRISMA: Preferred reporting items for systematic reviews and meta-analyses
RoB: Risk of bias
RT: Relaxation techniques
SLD: Specific learning disorders
SMD: Standardized mean differences
SUCRA: Surface under the cumulative ranking curve
UC: Usual care
