# Supplementary material for: Comparative effectiveness of physical activity interventions on cognitive functions in children and adolescents with Neurodevelopmental Disorders: a systematic review and network meta-analysis of randomized controlled trials
Source: Int J Behav Nutr Phys Act. 2025 Jan 13;22:6. doi: 10.1186/s12966-024-01702-7 (PMC11731537; doi:10.1186/s12966-024-01702-7)
Supplement: Supplementary file 1 — Supplementary Material 1. [file 12966_2024_1702_MOESM1_ESM.docx]

**SUPPLEMENTARY MATERIAL**

**Comparative effectiveness of physical activity interventions on cognitive functions in children and adolescents with Neurodevelopmental Disorders: a systematic review and network meta-analysis of randomised controlled trials**

Ruiyuan Tao^1^, Yijian Yang^1^, Mark Wilson^2^, Jeremy R. Chang^3^, Chang Liu^4^, Cindy H.P. Sit^1^

1. Department of Sports Science and Physical Education, The Chinese University of Hong Kong, Shatin, Hong Kong Special Administrative Region, China
2. Department of Public Health and Sports Sciences, Faculty of Health and Life Sciences, University of Exeter, Heavitree Road, Exeter, EX1 2LU, Devon, UK
3. Department of Rehabilitation Sciences, The Hong Kong Polytechnic University, Hong Kong SAR, China
4. Vanke School of Public Health, Tsinghua University, Beijing, China

Corresponding author: Prof. Cindy H.P. Sit, Department of Sports Science and Physical Education, The Chinese University of Hong Kong, Shatin, Hong Kong Special Administrative Region, China. Email: [sithp@cuhk.edu.hk](mailto:sithp@cuhk.edu.hk). Phone: +852 3943 4126

**Index of supplementary materia****l**

[Supplementary 1 PRISMA checklist 3](#_Toc180873005)

[Supplementary 2 Search strategy 6](#_Toc180873006)

[Supplementary 3 General characteristics of included studies 7](#_Toc180873007)

[Supplementary 4 Cochrane risk-of-bias assessment results 8](#_Toc180873008)

[Supplementary 5 Pairwise meta-analyses between PA interventions and usual care 9](#_Toc180873009)

[Supplementary 6 Assessment of transitivity 11](#_Toc180873010)

[Supplementary 7 Assessment of inconsistency 11](#_Toc180873011)

[Supplementary 8 Assessment of heterogeneity 14](#_Toc180873012)

[Supplementary 9 Network plot for acceptability 15](#_Toc180873013)

[Supplementary 10 League table for acceptability 15](#_Toc180873014)

[Supplementary 11 SUCRA results and ranking plots 16](#_Toc180873015)

[Supplementary 12 Comparison-adjusted funnel plots 18](#_Toc180873016)

[Supplementary 13 League tables for specific NDDs 20](#_Toc180873017)

[Supplementary 14 Sensitivity Analyses 22](#_Toc180873018)

[Supplementary 15 Meta-regression and subgroup analyses 24](#_Toc180873019)

[Supplementary 16 Grading the evidence of the network meta-analysis using CINeMA 27](#_Toc180873020)

[Reference 30](#_Toc180873021)

# Supplementary 1 PRISMA checklist

| **Section/Topic** | **Item #** | **Checklist Item** | **Reported**  **on Page #** |
| --- | --- | --- | --- |
| **TITLE** |  |  |  |
| Title | 1 | Identify the report as a systematic review *incorporating a network meta-analysis (or related form of meta-analysis)*. | 1 |
| **ABSTRACT** |  |  |  |
| Structured summary | 2 | Provide a structured summary including, as applicable:   - **Background:** main objectives - **Methods:** data sources; study eligibility criteria, participants, and interventions; study appraisal; and *synthesis methods, such as network meta-analysis.* - **Results:** number of studies and participants identified; summary estimates with corresponding confidence/credible intervals; *treatment rankings may also be discussed. Authors may choose to summarize pairwise comparisons against a chosen treatment included in their analyses for brevity.* - **Discussion/Conclusions:** limitations; conclusions and implications of findings. - **Other:** primary source of funding; systematic review registration number with registry name. | 2 |
| **INTRODUCTION** |  |  |  |
| Rationale | 3 | Describe the rationale for the review in the context of what is already known*, including mention of why a network meta-analysis has been conducted.* | 4-5 |
| Objectives | 4 | Provide an explicit statement of questions being addressed, with reference to participants, interventions, comparisons, outcomes, and study design (PICOS). | 5 |
| **METHODS** |  |  |  |
| Protocol and registration | 5 | Indicate whether a review protocol exists and if and where it can be accessed (e.g., Web address); and, if available, provide registration information, including registration number. | 5 |
| Eligibility criteria | 6 | Specify study characteristics (e.g., PICOS, length of follow-up) and report characteristics (e.g., years considered, language, publication status) used as criteria for eligibility, giving rationale. *Clearly describe eligible treatments included in the treatment network, and note whether any have been clustered or merged into the same node (with justification).* | 5-6 |
| Information sources | 7 | Describe all information sources (e.g., databases with dates of coverage, contact with study authors to identify additional studies) in the search and date last searched. | 5 |
| Search | 8 | Present full electronic search strategy for at least one database, including any limits used, such that it could be repeated. | 5, Supplementary 2 |
| Study selection | 9 | State the process for selecting studies (i.e., screening, eligibility, included in systematic review, and, if applicable, included in the meta-analysis). | 5-7 |
| Data collection process | 10 | Describe method of data extraction from reports (e.g., piloted forms, independently, in duplicate) and any processes for obtaining and confirming data from investigators. | 7 |
| Data items | 11 | List and define all variables for which data were sought (e.g., PICOS, funding sources) and any assumptions and simplifications made. | 5-7 |
| **Geometry of the network** | **S1** | Describe methods used to explore the geometry of the treatment network under study and potential biases related to it. This should include how the evidence base has been graphically summarized for presentation, and what characteristics were compiled and used to describe the evidence base to readers. | 7-8 |
| Risk of bias within individual studies | 12 | Describe methods used for assessing risk of bias of individual studies (including specification of whether this was done at the study or outcome level), and how this information is to be used in any data synthesis. | 7 |
| Summary measures | 13 | State the principal summary measures (e.g., risk ratio, difference in means). *Also describe the use of additional summary measures assessed, such as treatment rankings and surface under the cumulative ranking curve (SUCRA) values, as well as modified approaches used to present summary findings from meta-analyses.* | 7-8 |
| Planned methods of analysis | 14 | Describe the methods of handling data and combining results of studies for each network meta-analysis. This should include, but not be limited to:   - *Handling of multi-arm trials;* - *Selection of variance structure;* - *Selection of prior distributions in Bayesian analyses; and* - *Assessment of model fit.* | 7-8 |
| **Assessment of Inconsistency** | **S2** | Describe the statistical methods used to evaluate the agreement of direct and indirect evidence in the treatment network(s) studied. Describe efforts taken to address its presence when found. | 8 |
| Risk of bias across studies | 15 | Specify any assessment of risk of bias that may affect the cumulative evidence (e.g., publication bias, selective reporting within studies). | 7 |
| Additional analyses | 16 | Describe methods of additional analyses if done, indicating which were pre-specified. This may include, but not be limited to, the following:   - Sensitivity or subgroup analyses; - Meta-regression analyses; - *Alternative formulations of the treatment network; and* - *Use of alternative prior distributions for Bayesian analyses (if applicable).* | 8 |
| **RESULTS†** |  |  |  |
| Study selection | 17 | Give numbers of studies screened, assessed for eligibility, and included in the review, with reasons for exclusions at each stage, ideally with a flow diagram. | 8-9, Fig. 1 |
| **Presentation of network structure** | **S3** | Provide a network graph of the included studies to enable visualization of the geometry of the treatment network. | Fig. 2, Supplementary 9 |
| **Summary of network geometry** | **S4** | Provide a brief overview of characteristics of the treatment network. This may include commentary on the abundance of trials and randomized patients for the different interventions and pairwise comparisons in the network, gaps of evidence in the treatment network, and potential biases reflected by the network structure. | 10-11,  Supplementary 10, 13 |
| Study characteristics | 18 | For each study, present characteristics for which data were extracted (e.g., study size, PICOS, follow-up period) and provide the citations. | 8, Supplementary 3 |
| Risk of bias within studies | 19 | Present data on risk of bias of each study and, if available, any outcome level assessment. | 13, Supplementary 4 |
| Results of individual studies | 20 | For all outcomes considered (benefits or harms), present, for each study: 1) simple summary data for each intervention group, and 2) effect estimates and confidence intervals. *Modified approaches may be needed to deal with information from larger networks.* | 10-12, Supplementary 6-16 |
| Synthesis of results | 21 | Present results of each meta-analysis done, including confidence/credible intervals. *In larger networks, authors may focus on comparisons versus a particular comparator (e.g. placebo or standard care), with full findings presented in an appendix. League tables and forest plots may be considered to summarize pairwise comparisons.* If additional summary measures were explored (such as treatment rankings), these should also be presented. | 8-12, Supplementary 6-16 |
| **Exploration for inconsistency** | **S5** | Describe results from investigations of inconsistency. This may include such information as measures of model fit to compare consistency and inconsistency models, *P* values from statistical tests, or summary of inconsistency estimates from different parts of the treatment network. | 9-10, Supplementary 7 |
| Risk of bias across studies | 22 | Present results of any assessment of risk of bias across studies for the evidence base being studied. | 13, Supplementary 16 |
| Results of additional analyses | 23 | Give results of additional analyses, if done (e.g., sensitivity or subgroup analyses, meta-regression analyses*, alternative network geometries studied, alternative choice of prior distributions for Bayesian analyses,* and so forth). | 13, Supplementary 14-15 |
| **DISCUSSION** |  |  |  |
| Summary of evidence | 24 | Summarize the main findings, including the strength of evidence for each main outcome; consider their relevance to key groups (e.g., healthcare providers, users, and policy-makers). | 13-15 |
| Limitations | 25 | Discuss limitations at study and outcome level (e.g., risk of bias), and at review level (e.g., incomplete retrieval of identified research, reporting bias). *Comment on the validity of the assumptions, such as transitivity and consistency. Comment on any concerns regarding network geometry (e.g., avoidance of certain comparisons).* | 15-16 |
| Conclusions | 26 | Provide a general interpretation of the results in the context of other evidence, and implications for future research. | 16 |
| **FUNDING** |  |  |  |
| Funding | 27 | Describe sources of funding for the systematic review and other support (e.g., supply of data); role of funders for the systematic review. This should also include information regarding whether funding has been received from manufacturers of treatments in the network and/or whether some of the authors are content experts with professional conflicts of interest that could affect use of treatments in the network. | 17 |

**Abbreviation:** PICOS = Population, Intervention, Comparators, Outcomes, Study design.

* Text in italics indicates wording specific to reporting of network meta-analyses that has been added to guidance from the PRISMA statement.

† Authors may wish to plan for use of appendices to present all relevant information in full detail for items in this section.

# Supplementary 2 Search strategy

1. AB=neurodevelopmental disorder*

2. AB=(“attention-deficit hyperactivity disorder*” or ADHD or “attention-deficit disorder*” or ADD or autism or autism spectrum disorder* or ASD or autistic or “communication disorder*” or “intellectual disabilit*” or “learning disabilit*” or “developmental coordination disorder*” or DCD)

3. #2 OR #1

4. AB=( child* or adolescent or teen* or youth* or school or student)

5. #4 AND #3

6. AB=(“physical activity” or exercise or training or sport or motor or “physical fitness” or aerobic or endurance or resistance or strength or HIIT or exergaming or gymnastics or running or cycling or ball or racket or treadmill or swimming or “martial arts” or mind-body or danc*)

7. AB=(cogniti* or “cognitive function*” or neurocogniti* or neuropsycholog*)

8. AB= (attention or “attentional control” or concentration or vigilance or memory or “executive function*” or “executive dysfunction*” or reasoning or “problem solving” or “executive control” or inhibit* or “impulse control” or “interference control” or “cognitive control” or self-control or “cognitive flexibility” or “mental flexibility” or shifting or switching or updating or “decision making” or planning or accuracy or “reaction time”)

9. #8 OR #7

10. TS=(random* or “random* controlled trial” or RCT)

11. #10 AND #9 AND #6 AND #5 (Limiters: Human, English)

# Supplementary 3 General characteristics of included studies

| **Characteristics** | **Studies (n=31)**  **Arms (n=66)** |
| --- | --- |
| **Total participants at baseline**, n | 1, 403 |
| **Age of participants** (year), mean±SD | 10.0±1.9 |
| **% of male participants,** mean | 83.2±16.1 |
| **NDDs Category**, n (% of studies) | |
| ADHD | 18 (58.1) |
| ASD | 10 (32.3) |
| DCD | 2 (6.4) |
| SLD | 1 (3.2) |
| **Continent**, n (% of studies) | |
| Africa | 3 (9.7) |
| Asia | 20 (64.5) |
| Europe | 5 (16.1) |
| North America | 2 (6.4) |
| South America | 1 (3.2) |
| **Treatment**, n (% of arms) |  |
| AE | 7 (10.6) |
| Exergaming | 5 (7.6) |
| MBE | 6 (9.1) |
| MPA | 19 (28.8) |
| NF | 3 (4.5) |
| RT | 1 (1.5) |
| UC | 27 (40.9) |
| **Outcome measures**, n (% of studies) | |
| Attention | 2 (6.4) |
| Memory | 1 (3.2) |
| Executive functions | 14 (45.2) |
| Attention & Memory | 2 (6.4) |
| Attention & Executive Functions | 3 (9.7) |
| Memory & Executive Functions | 6 (19.4) |
| Attention & Memory & Executive Functions | 3 (9.7) |

*ADHD* attention-deficit/hyperactivity disorder, *ASD* autism spectrum disorder, *DCD* developmental coordination disorder, S*LD* specific learning disorder; *Y* yes, *N* no, *NI* no information; *IQR* Interquartile Range; *AE* aerobic exercise, *MBE* mind-body exercise, *MPA* multicomponent physical activity, *RT* relaxation techniques, *NF* neurofeedback, *UC* usual care.

# Supplementary 4 Cochrane risk-of-bias assessment results

Fig. S4.1 Risk of bias assessment for the included studies

|  | Randomization process | Deviations from intended interventions | Mising outcome data | Measurement of the outcome | Selection of the reported result | Overall Quality |
| --- | --- | --- | --- | --- | --- | --- |
| Ahmed & Mohamed (2011) | Some concerns | Some concerns | Low | High | Some concerns | High |
| Benzing & Schmidt (2019) | Low | Some concerns | Low | Low | Low | Some concerns |
| Borgi et al. (2016) | Some concerns | Some concerns | Low | Low | Some concerns | Some concerns |
| Bustamante et al. (2016) | Some concerns | Some concerns | Low | Low | Some concerns | Some concerns |
| Chan et al. (2013) | Low | Some concerns | Low | Low | Some concerns | Some concerns |
| Chang et al. (2022) | Low | Some concerns | Low | Low | Low | Some concerns |
| Choi et al. (2015) | Some concerns | Some concerns | Some concerns | Low | Some concerns | Some concerns |
| Damanpak & Sabzi (2022) | Some concerns | Some concerns | Low | High | Some concerns | High |
| Emami Kashfi et al. (2019) | Some concerns | Some concerns | Low | Low | Some concerns | Some concerns |
| Geladé et al. (2017) | Low | Some concerns | Low | Low | Some concerns | Some concerns |
| Greco & De Ronzi (2020) | Low | Some concerns | Low | High | Some concerns | High |
| Hashemi et al. (2022) | Low | Low | Low | Some concerns | Some concerns | Some concerns |
| Hattabi et al. (2019) | Some concerns | Some concerns | Low | Low | Some concerns | Some concerns |
| Ji & Yang (2022) | Some concerns | High | High | Low | Some concerns | High |
| Ji et al. (2023) | Some concerns | Some concerns | Some concerns | Some concerns | Low | Some concerns |
| Kadri et al. (2019) | Some concerns | Some concerns | Low | Low | Some concerns | Some concerns |
| Lee et al. (2017) | Some concerns | Some concerns | Some concerns | Low | Some concerns | Some concerns |
| Liang et al. (2022) | Low | Some concerns | Low | Low | Some concerns | Some concerns |
| Ludyga et al. (2022) | Low | Some concerns | Low | Low | Some concerns | Some concerns |
| Memarmoghaddam et al. (2016) | Some concerns | Some concerns | Low | Low | Some concerns | Some concerns |
| Nejati & Derakhshan (2021) | Some concerns | Some concerns | Low | Low | Some concerns | Some concerns |
| Pan et al. (2016) | Some concerns | Some concerns | Low | Low | Some concerns | Some concerns |
| Pan et al. (2017) | Some concerns | Some concerns | Low | Low | Some concerns | Some concerns |
| Phung & Goldberg (2019) | Some concerns | Some concerns | Low | Low | Some concerns | Some concerns |
| Rafiei Milajerdi et al. (2021) | Some concerns | Low | Low | Low | Some concerns | Some concerns |
| Rezaei et al. (2018) | Some concerns | Some concerns | Low | Low | Some concerns | Some concerns |
| Sani et al. (2022) | Some concerns | Some concerns | High | Low | Some concerns | High |
| Silva et al. (2020) | Some concerns | Some concerns | Some concerns | Low | Some concerns | Some concerns |
| Tse et al. (2019) | Some concerns | Some concerns | Low | Low | Some concerns | Some concerns |
| Tse et al. (2021) | Some concerns | Some concerns | Low | Low | Some concerns | Some concerns |
| Tse et al. (2023) | Some concerns | Some concerns | Low | Low | Some concerns | Some concerns |

Fig. S4.2 Aggregate results


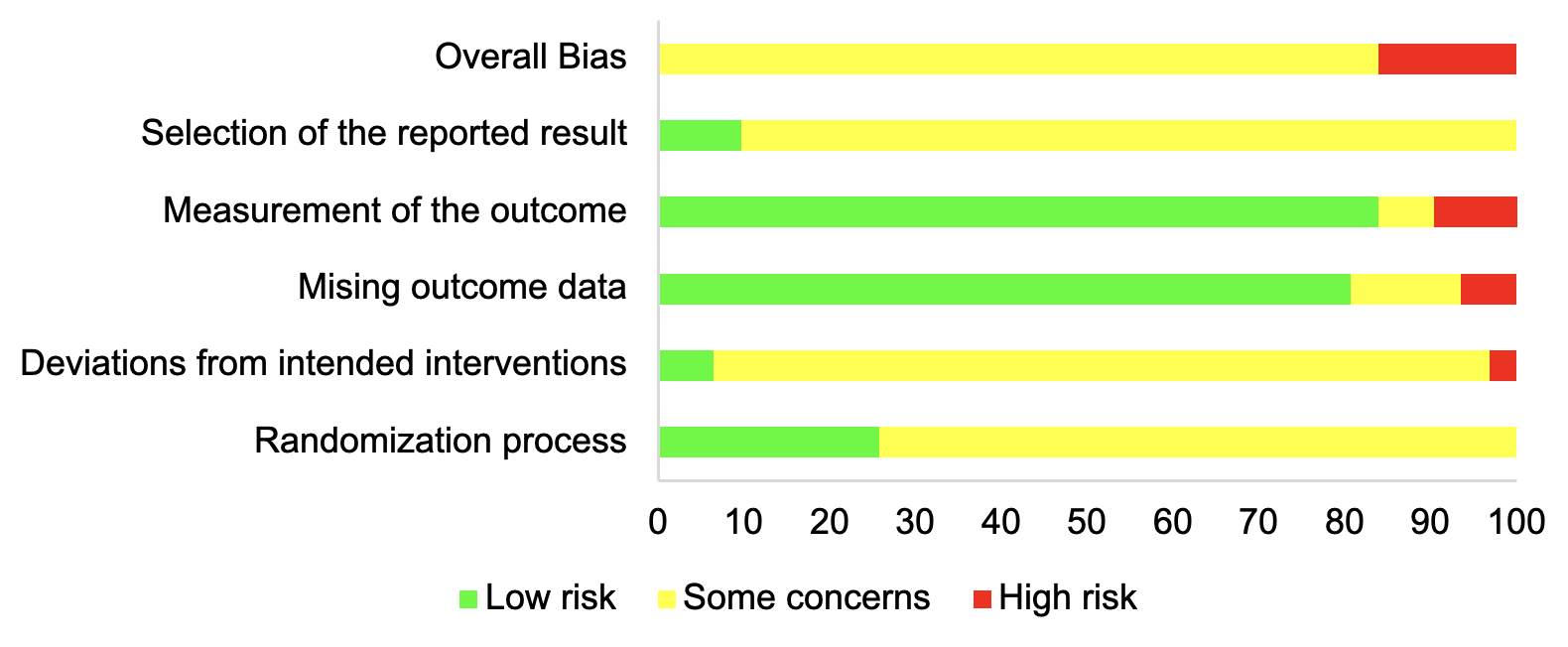


# Supplementary 5 Pairwise meta-analyses between PA interventions and usual care

| **Treatment 1** | **Treatment 2** | **Outcome** | ***N* of**  **comparisons** | **Hedges’ *g*** | **95%CI** | **I^2^ (%)** |
| --- | --- | --- | --- | --- | --- | --- |
| Four types of PA interventions | Usual care | Attention | 7 | 1.263 | (0.435, 2.090) | 90.36 |
|  |  | Memory | 10 | 0.463 | (0.068, 0.858) | 71.38 |
|  |  | Executive functions | 24 | 0.798 | (0.466, 1.131) | 80.16 |
|  |  | **Outcome** | ***N* of**  **comparisons** | **Odds ratio** | **95%CI** | **I^2^ (%)** |
|  |  | Acceptabilitys | 28 | 0.883 | (0.527, 1.480) | 11.30 |

Table S5.1 Pairwise meta-analyses for attention

| **Treatment 1** | **Treatment 2** | **N of Studies** | **Hedges' *g*** | **95% CI** | **I^2^ (%)** |
| --- | --- | --- | --- | --- | --- |
| AE | NF | 1 | -0.171 | (-0.671, 0.330) | N/A |
| AE | UC | 1 | **0.496** | (0.066, 0.926) | N/A |
| Exergaming | AE | 1 | 0.121 | (-0.588, 0.820) | N/A |
| Exergaming | UC | 1 | **2.283** | (1.577, 2.988) | N/A |
| MBE | NF | 1 | **1.207** | (0.129, 2.285) | N/A |
| MBE | UC | 2 | 1.836 | (-0.267, 3.940) | 89.8 |
| MPA | NF | 1 | **0.894** | (0.255, 1.532) | N/A |
| MPA | UC | 3 | 0.819 | (-0.363, 2.002) | 87.7 |
| NF | UC | 1 | 0.116 | (-0.866, 1.098) | N/A |

Table S5.2 Pairwise meta-analyses for memory

| **Treatment 1** | **Treatment 2** | **N of Studies** | **Hedges' *g*** | **95% CI** | **I^2^ (%)** |
| --- | --- | --- | --- | --- | --- |
| AE | MPA | 2 | -0.657 | (-1.440, 0.126) | 62.8 |
| AE | NF | 1 | 0.058 | (-0.387, 0.503) | N/A |
| AE | UC | 2 | 0.032 | (-0.415, 0.478) | 0.0 |
| Exergaming | UC | 2 | 0.990 | (-0.560, 2.539) | 92.1 |
| MBE | NF | 1 | -0.053 | (-1.034, 0.928) | N/A |
| MBE | UC | 2 | **0.496** | (0.024, 0.967) | 2.1 |
| MPA | UC | 4 | 0.365 | (-0.218, 0.949) | 69.9 |
| NF | UC | 1 | **1.061** | (0.005, 2.118) | N/A |

Table S5.3 Pairwise meta-analyses for executive functions

| **Treatment 1** | **Treatment 2** | **N of Studies** | **Hedges' *g*** | **95% CI** | **I^2^ (%)** |
| --- | --- | --- | --- | --- | --- |
| AE | MPA | 3 | **-0.767** | (-1.192, -0.341) | 17.8 |
| AE | NF | 1 | -0.114 | (-0.581, 0.353) | N/A |
| AE | UC | 2 | 0.067 | (-0.379, 0.514) | 0.0 |
| Exergaming | AE | 1 | **0.927** | (0.191, 1.664) | N/A |
| Exergaming | MPA | 1 | -0.546 | (-1.225, 0.133) | N/A |
| Exergaming | UC | 4 | **0.870** | (0.064, 1.676) | 83.8 |
| MBE | RT | 1 | 0.533 | (-0.093, 1.159) | N/A |
| MBE | UC | 3 | 0.934 | (-0.132, 2.000) | 84.8 |
| MPA | AE | 1 | 0.379 | (-0.222, 0.981) | N/A |
| MPA | UC | 15 | **0.856** | (0.419, 1.294) | 82.5 |

# Supplementary 6 Assessment of transitivity

Table S6 General characteristics of treatment nodes

| **Nodes** | **N of arms**  **(%)** | **N of participants**  **(%)** | **Mean age**  **(SD)** | **Mean percentage**  **of male (SD)** | **Mean treatment length**  **(weeks, SD)** | **Mean treatment dose**  **(minutes, SD)** |
| --- | --- | --- | --- | --- | --- | --- |
| AE | 7 (10.6) | 179 (12.8) | 10.1 (1.5) | 75.7 (8.9) | 5.9 (3.6) | 1155 (876.3) |
| Exergaming | 5 (7.6) | 110 (7.8) | 9.1 (1.0) | 90.1 (7.4) | 8.0 (2.8) | 1008 (649.6) |
| MBE | 6 (9.1) | 109 (7.8) | 10.63 (2.1) | 86.6 (14.7) | 21.2 (28.0) | 2155 (2789) |
| MPA | 19 (28.8) | 390 (27.8) | 9.7 (2.1) | 85.4 (18.7) | 8.3 (3.4) | 1444 (707.3) |
| NF | 3 (4.5) | 71 (5.1) | 8.8 (1.3) | 71.0 (8.4) | 8.0 (1.0) | 1063 (205.5) |
| RT | 1 (1.5) | 23 (1.6) | 12.4 | 85.0 | 4.0 | 360 |
| UC | 27 (40.9) | 521 (37.1) | 10.1 (2.1) | 83.2 (18.2) | 12.1 (14.0) | 1747 (1433) |
| Total/Mean | 66 | 1,403 | 10.0 (1.9) | 83.5 (16.3) | 10.6 (12.4) | 1526.2 (1310.1) |
| Q1 |  |  | 8.7 | 75.4 | 6.0 | 720.0 |
| Q2 |  |  | 9.5 | 86.0 | 8.0 | 1215.0 |
| Q3 |  |  | 10.4 | 100.0 | 12.0 | 1680.0 |

AE=aerobic exercise, MBE=mind-body exercise, MPA= multicomponent physical activity, RT=relaxation techniques, NF=neurofeedback, UC=usual care; Q1=first quartile, Q2=second quartile, Q3=third quartile.

Number of studies that are unavailable for analysis: gender (n=2).

# Supplementary 7 Assessment of inconsistency

Table S7.1 Estimated global inconsistency in networks

| **Outcomes** | **Chi square** | **Prob>Chi2** |
| --- | --- | --- |
| Attention | Chi2 (4) = 3.47 | Prob>Chi2 = 0.482 |
| Memory | Chi2 (5) = 1.03 | Prob>Chi2 = 0.960 |
| Executive functions | Chi2 (6) = 1.68 | Prob>Chi2 = 0.947 |
| Acceptability | Chi2 (9) = 6.28 | Prob>Chi2 = 0.712 |

Table S7.2 Estimated local inconsistency for each pairwise comparison (side-splitting)

Table S7.2a Estimated local inconsistency for attention


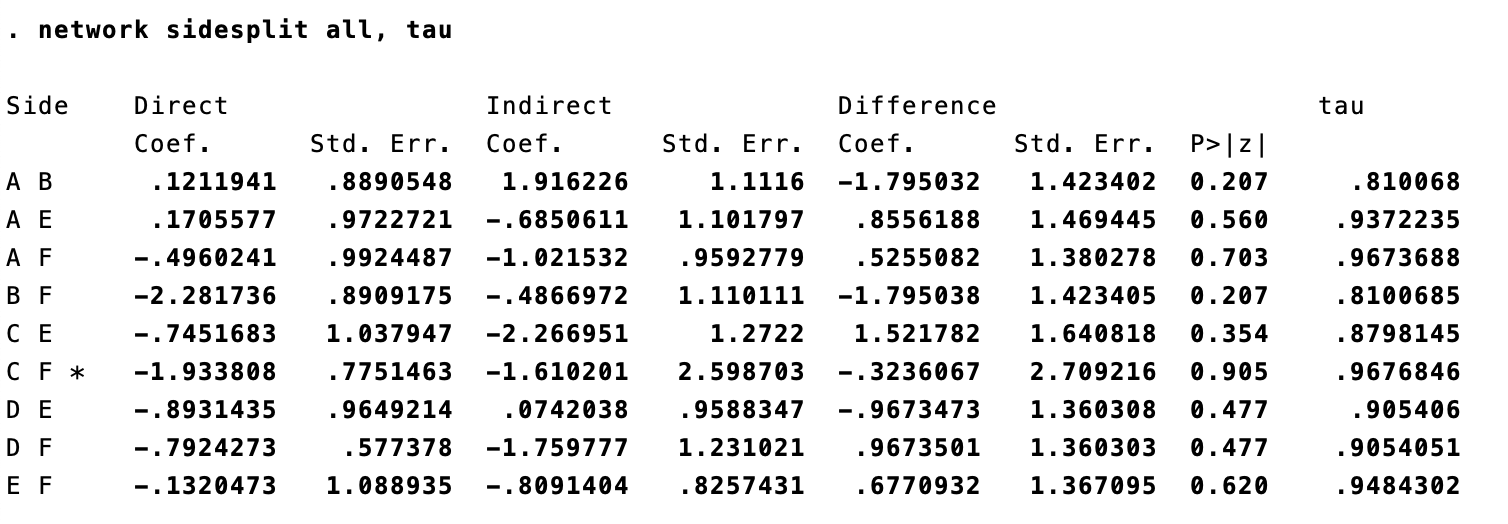


Table S7.2b Estimated local inconsistency for memory


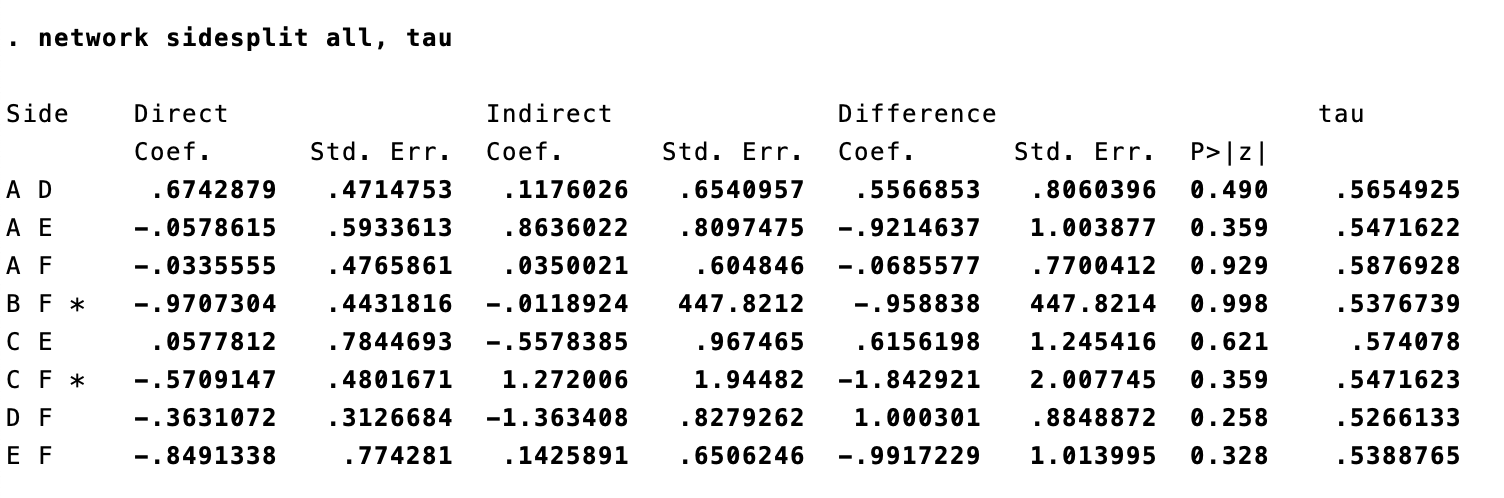


Table S7.2c Estimated local inconsistency for executive functions


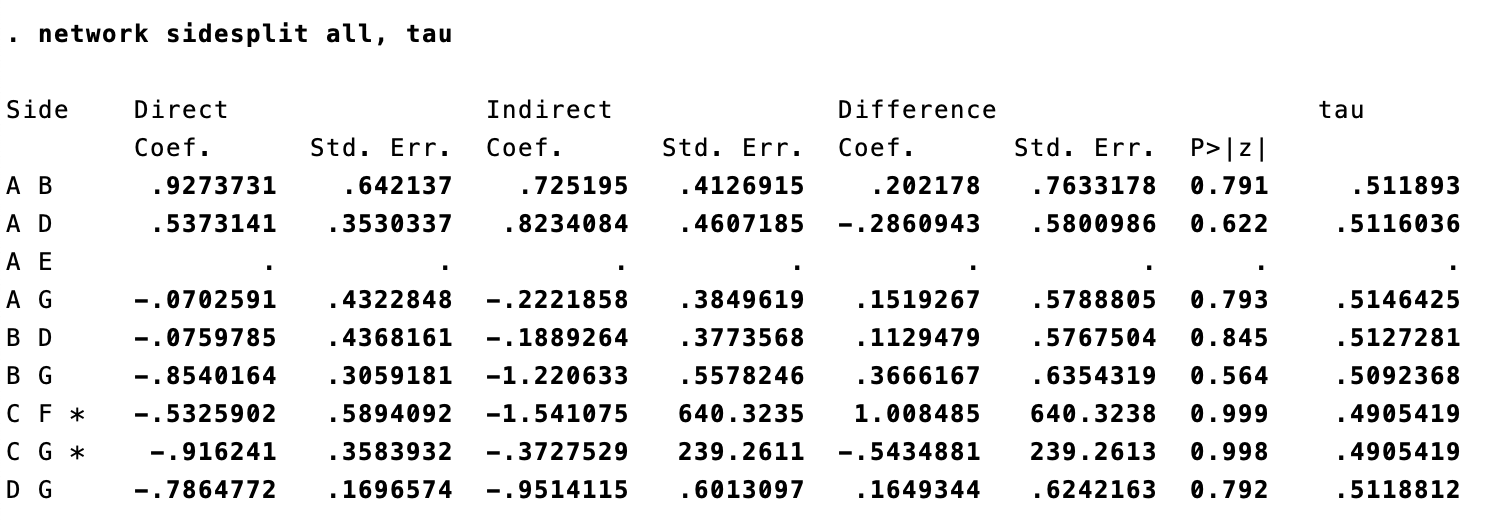


Table S7.2d Estimated local inconsistency for acceptability


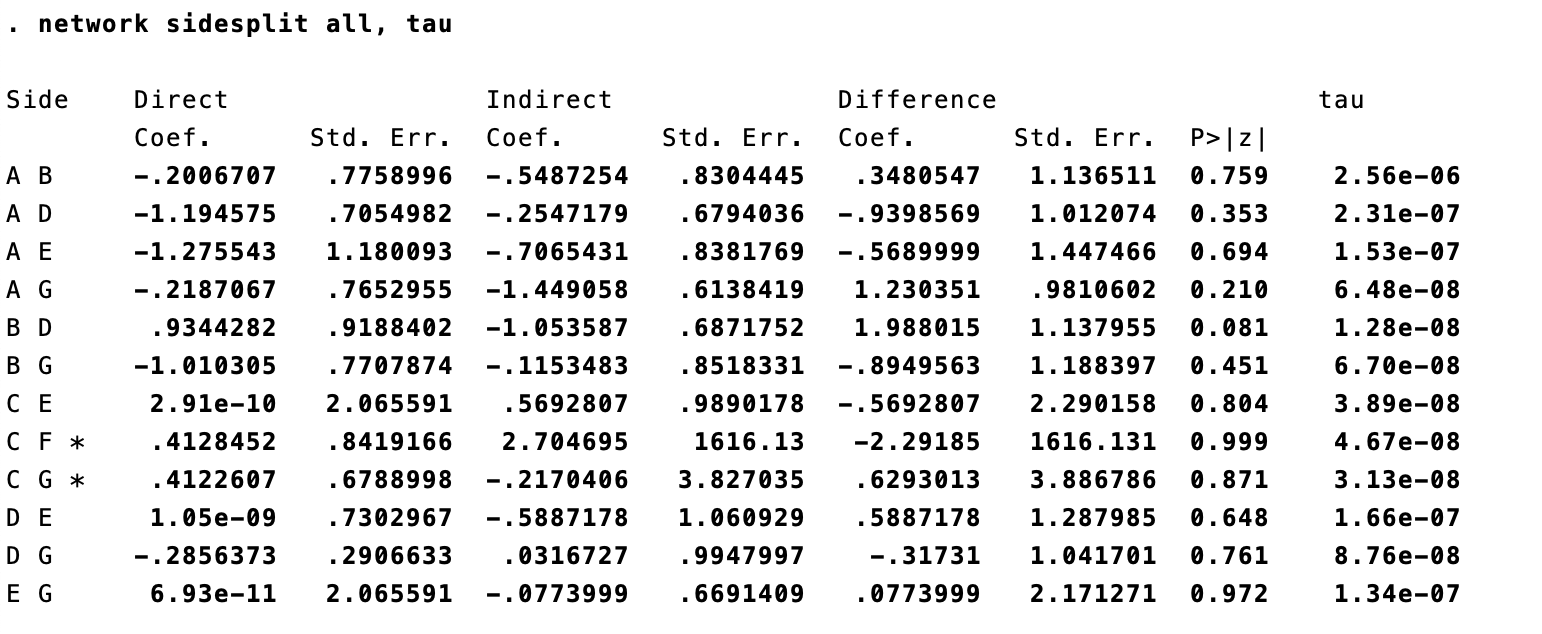


# Supplementary 8 Assessment of heterogeneity

The heterogeneity of entire NMA models was estimated by a common heterogeneity variance (**τ^2^**), which was compared with empirical distributions for continuous [1] and for dichotomous data [2]. Heterogeneity was quantified as low (from 0.01 to 0.025), moderate (>0.025 to 1.0), and high (>1.0) [3].

Table S8.1 Continuous outcomes

| **Continuous Outcome** | **Between study variance**  **(****τ^2^)** | **Outcome types**  **used as comparator** | **Predictive distribution of**  **τ^2^ Median (95%CI)** |
| --- | --- | --- | --- |
| Attention | 0.681 | Mental health outcome | 0.058 (0.001, 2.58) |
| Memory | 0.317 | Mental health outcome | 0.058 (0.001, 2.58) |
| Executive Functions | 0.277 | Mental health outcome | 0.058 (0.001, 2.58) |

Table S8.2 Dichotomous outcome

| **Dichotomous outcomes** | **Between study variance**  **(τ^2^)** | **Outcome types used**  **as comparator** | **Predictive distribution of**  **τ^2^ Median (95%CI)** |
| --- | --- | --- | --- |
| Acceptability | 0.084 | Withdrawals | 0.12 (0.08, 0.16) |

# Supplementary 9 Network plot for acceptability


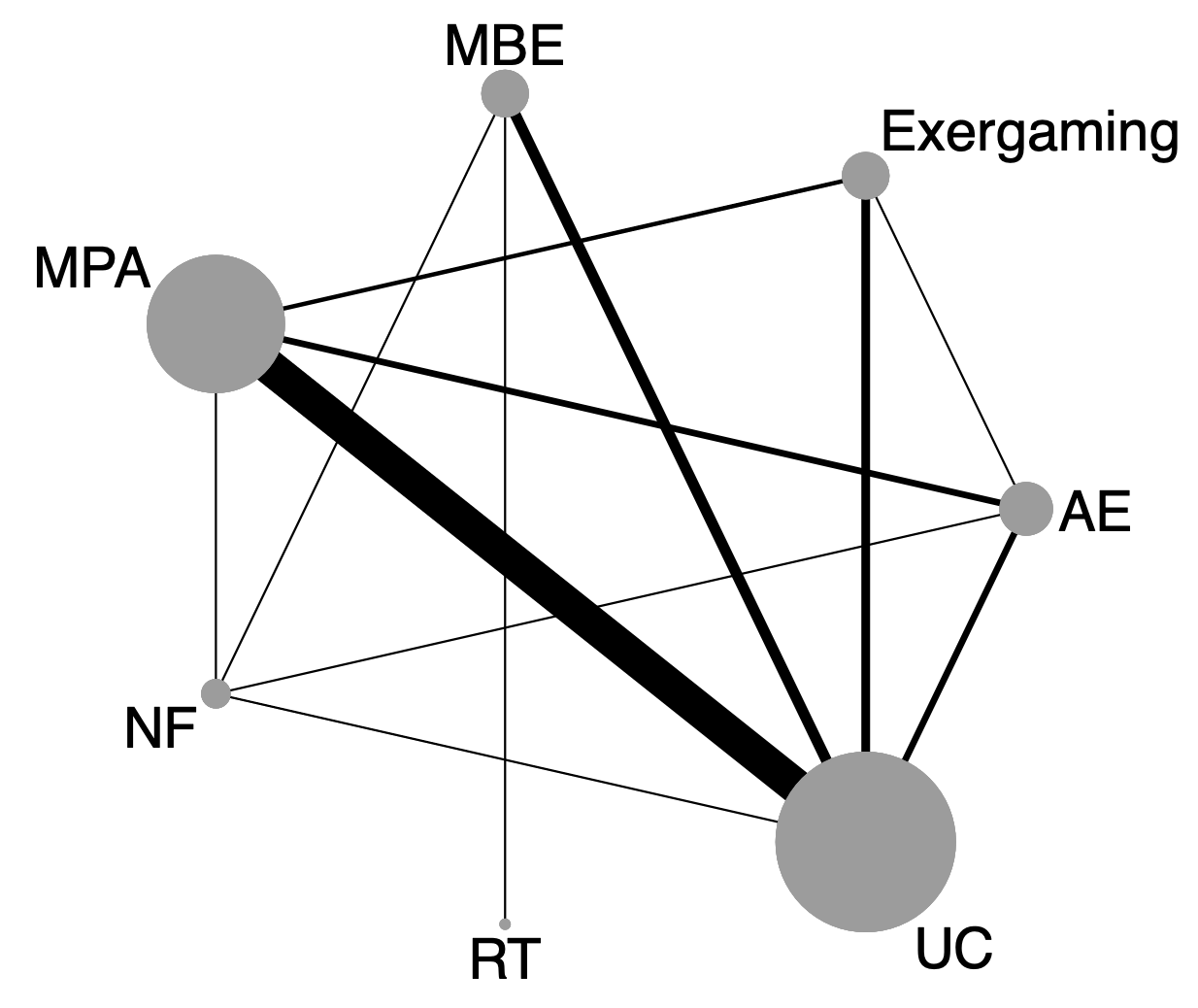


Each node represents a treatment. Connecting lines between two nodes represent one or more RCTs in which the two nodes have been compared directly. Size of each node indicates the number of participants. Thickness of the lines indicates the number of RCTs that directly compared the treatments it connected. *AE* aerobic exercise, *MBE* mind-body exercise, *MPA* multicomponent physical activity, *RT* relaxation techniques, *NF* neurofeedback, *UC* usual care.

# Supplementary 10 League table for acceptability

| MBE |  |  |  |  |  |  |
| --- | --- | --- | --- | --- | --- | --- |
| 0.67 (0.18,2.50) | UC |  |  |  |  |  |
| 0.63 (0.11,3.62) | 0.93 (0.27,3.25) | NF |  |  |  |  |
| 0.66 (0.13,3.45) | 0.98 (0.12,8.06) | 1.05 (0.10,11.64) | RT |  |  |  |
| 0.52 (0.13,2.13) | 0.77 (0.45,1.33) | 0.83 (0.25,2.69) | 0.79 (0.09,6.88) | MPA |  |  |
| 0.37 (0.07,2.00) | 0.55 (0.19,1.61) | 0.59 (0.13,2.71) | 0.56 (0.05,5.91) | 0.71 (0.24,2.10) | Exergaming |  |
| 0.26 (0.05,1.27) | **0.38 (0.15,0.97)** | 0.41 (0.11,1.56) | 0.39 (0.04,3.85) | 0.49 (0.20,1.24) | 0.70 (0.23,2.11) | AE |

*AE* aerobic exercise, *MBE* mind-body exercise, *MPA* multicomponent physical activity, *RT* relaxation techniques, *NF* neurofeedback, *UC* usual care. Results are presented as odds ratio (OR) and 95% confidence intervals. OR values less than 1.00 favor the column-defining treatment node for the NMA results. Estimates in bold denote significance at *p*<0.05.

# Supplementary 11 SUCRA results and ranking plots

Table S11 SUCRA results

|  | Attention | Memory | Executive Functions | Acceptability |
| --- | --- | --- | --- | --- |
| AE | 41.7 | 22.3 | 26.0 | 12.0 |
| Exergaming | 72.3 | 88.0 | 80.6 | 30.3 |
| MBE | 83.4 | 59.2 | 78.1 | 79.1 |
| MPA | 55.2 | 64.3 | 70.2 | 47.1 |
| NF | 40.7 | 45.9 | 37.1 | 59.2 |
| RT | N/A | N/A | 42.7 | 55.0 |
| UC | 6.6 | 20.3 | 15.3 | 67.3 |

*AE* aerobic exercise, *MBE* mind-body exercise, *MPA* multicomponent physical activity, *RT* relaxation techniques, *NF* neurofeedback, *UC* usual care. *SUCRA* surface under the cumulative ranking curve, *N/A* not applicable. SCURA in bold denotes ranked as the first effective treatment for each outcome.

Fig. S11 Ranking plots

*AE* aerobic exercise, *MBE* mind-body exercise, *MPA* multicomponent physical activity, *RT* relaxation techniques, *NF* neurofeedback, *UC* usual care.


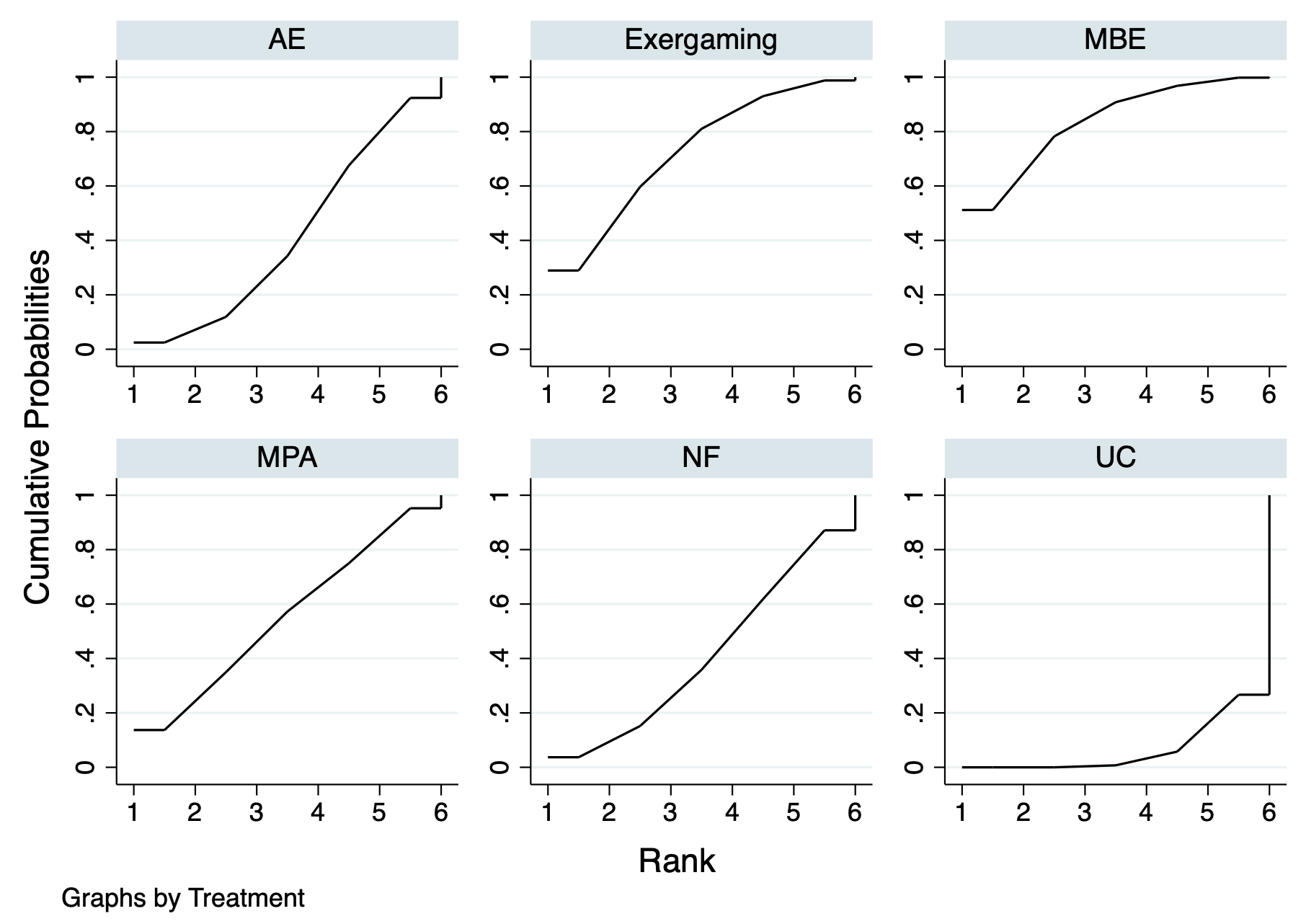


Fig. S11.1 Ranking plot for attention


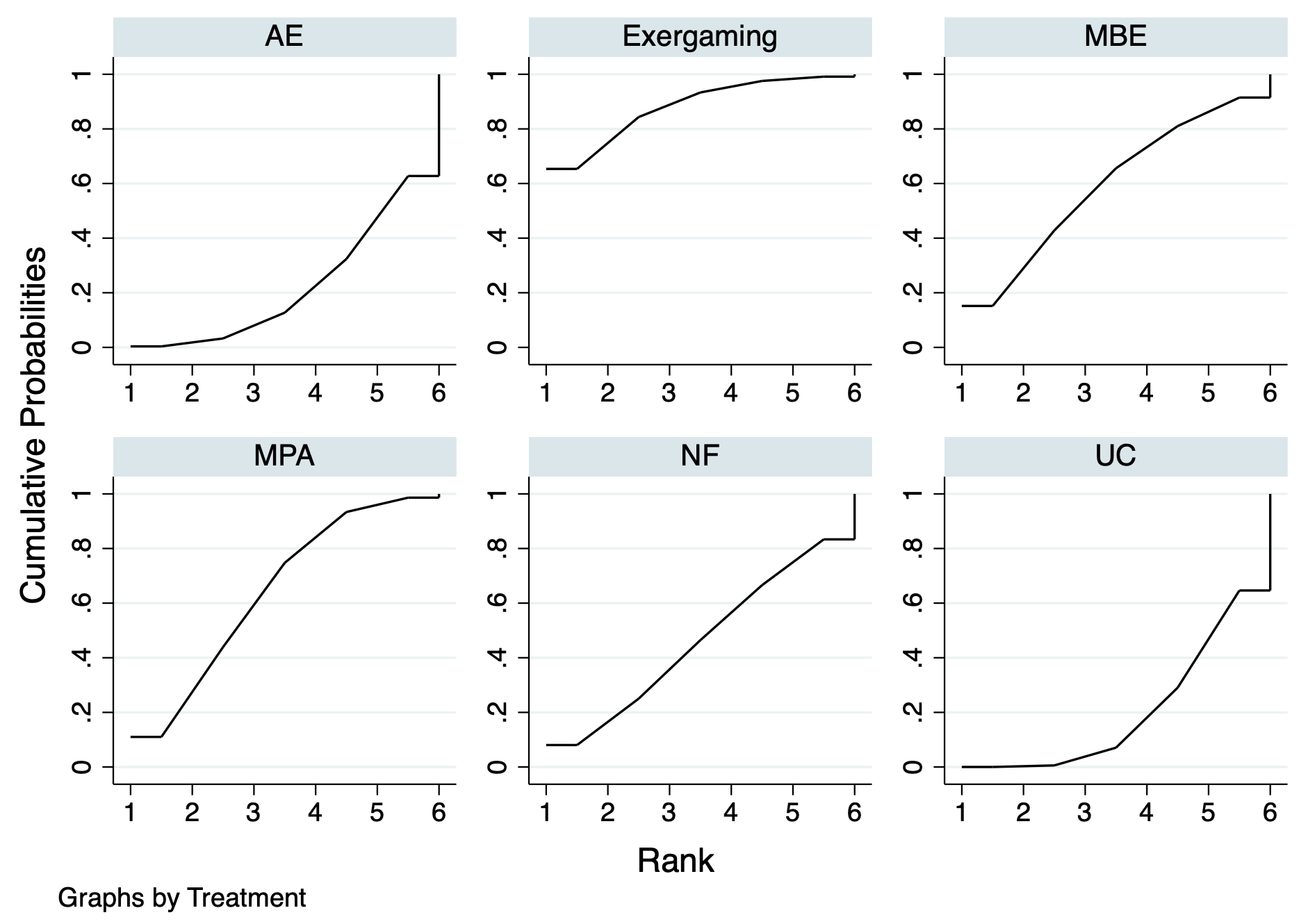


Fig. S11.2 Ranking plot for memory


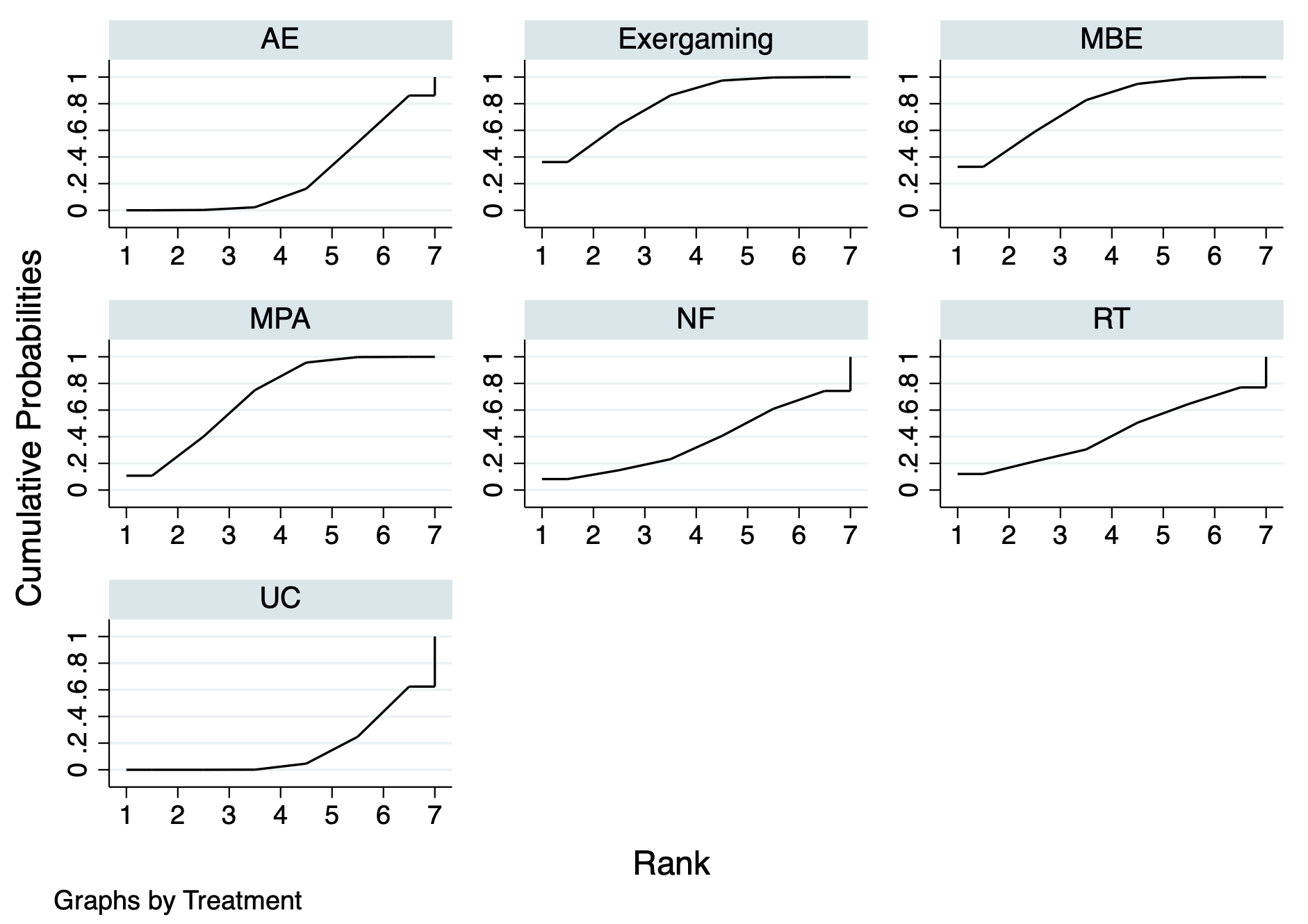


Fig. S11.3 Ranking plot for executive functions


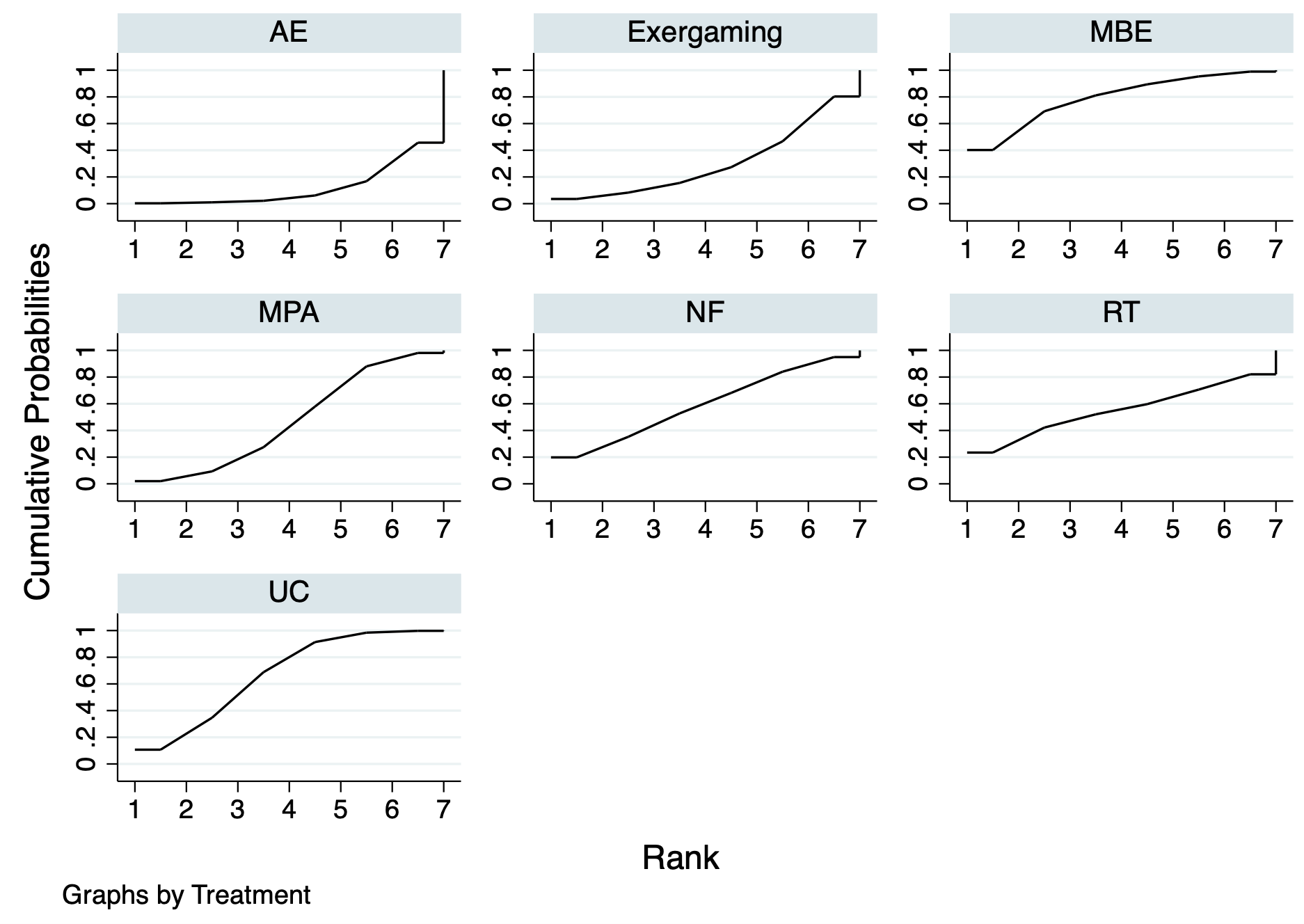


Fig. S11.4 Ranking plot for acceptability

# Supplementary 12 Comparison-adjusted funnel plots

Given that no studies evaluating memory were classified as having a high risk of bias, sensitivity analysis for memory was not performed.


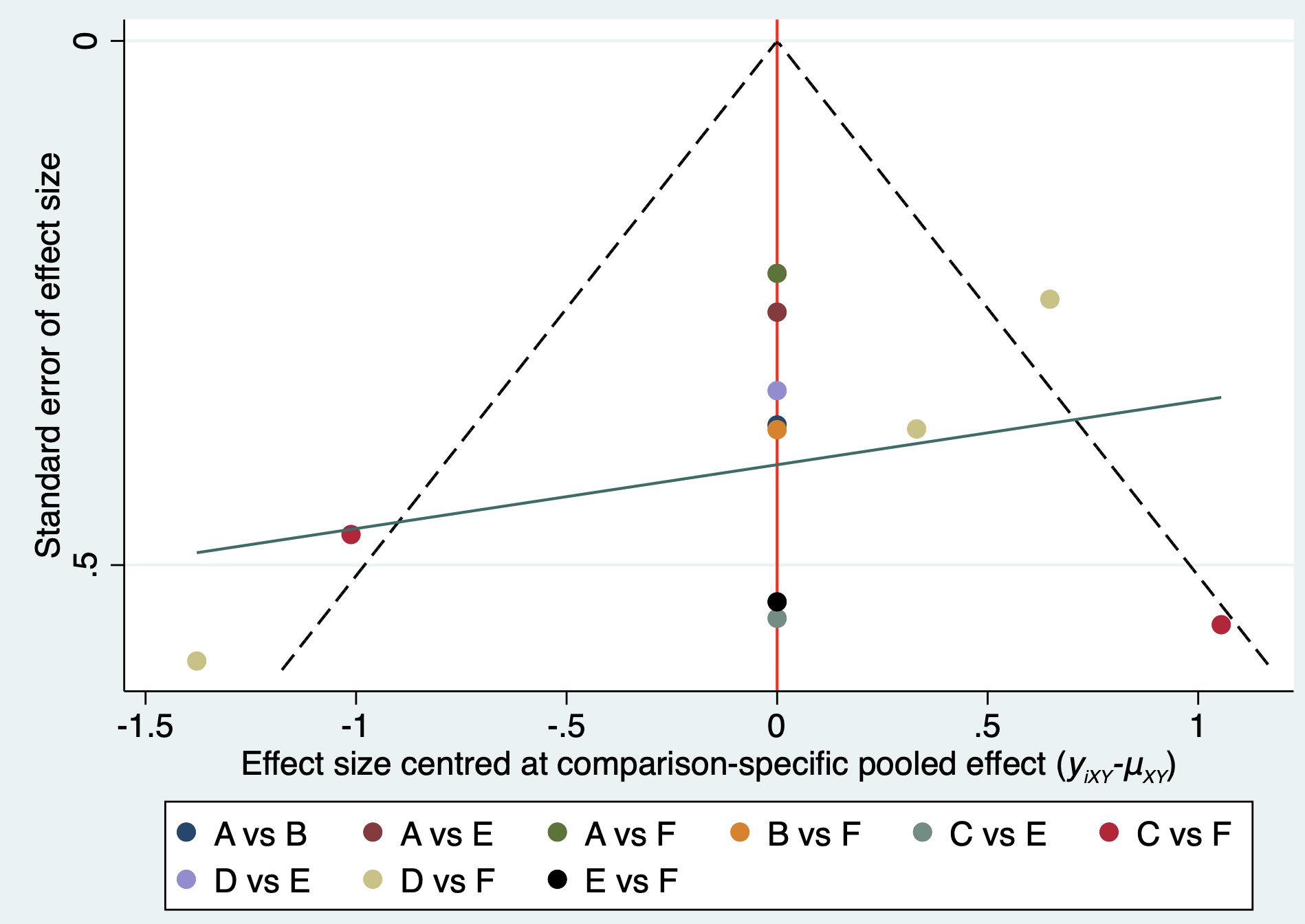


Fig. S12.1 Comparison-adjusted funnel plots for attention (Egger test: *p* = 0.304)


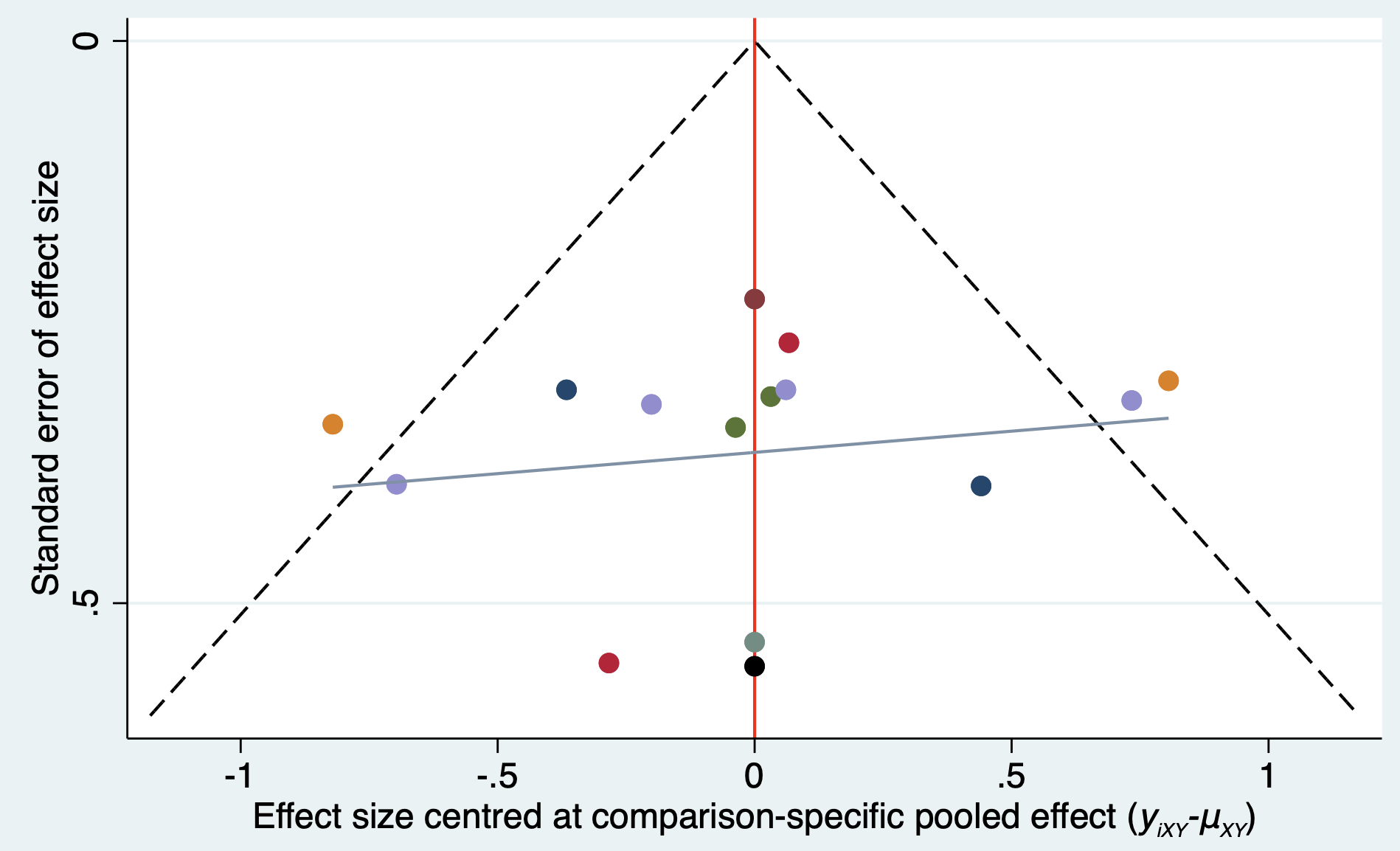


Fig. S12.2 Comparison-adjusted funnel plots for memory (Egger test: p = 0.585)


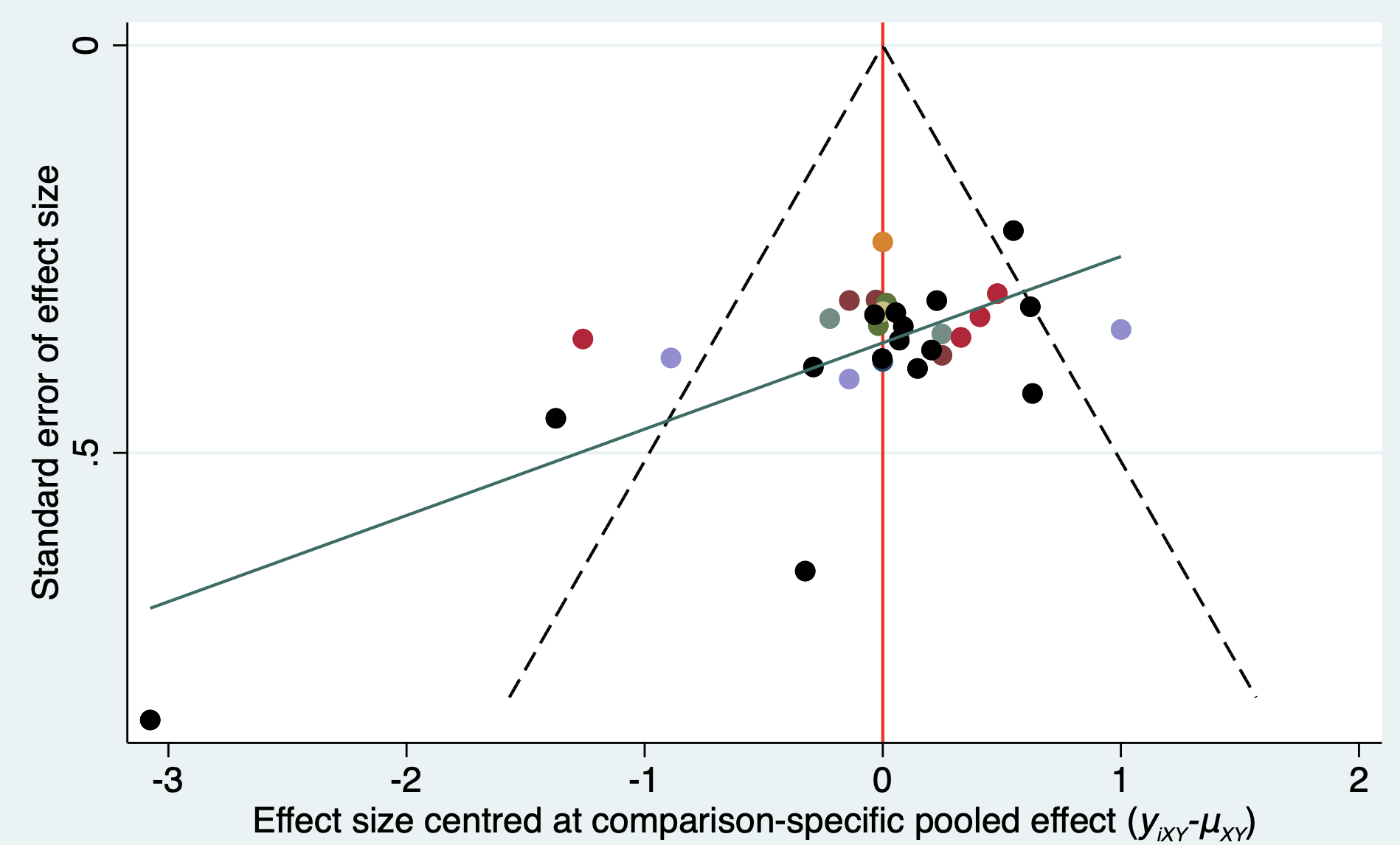


Fig. S12.3 Comparison-adjusted funnel plots for executive functions (Egger test: *p* = 0.003)


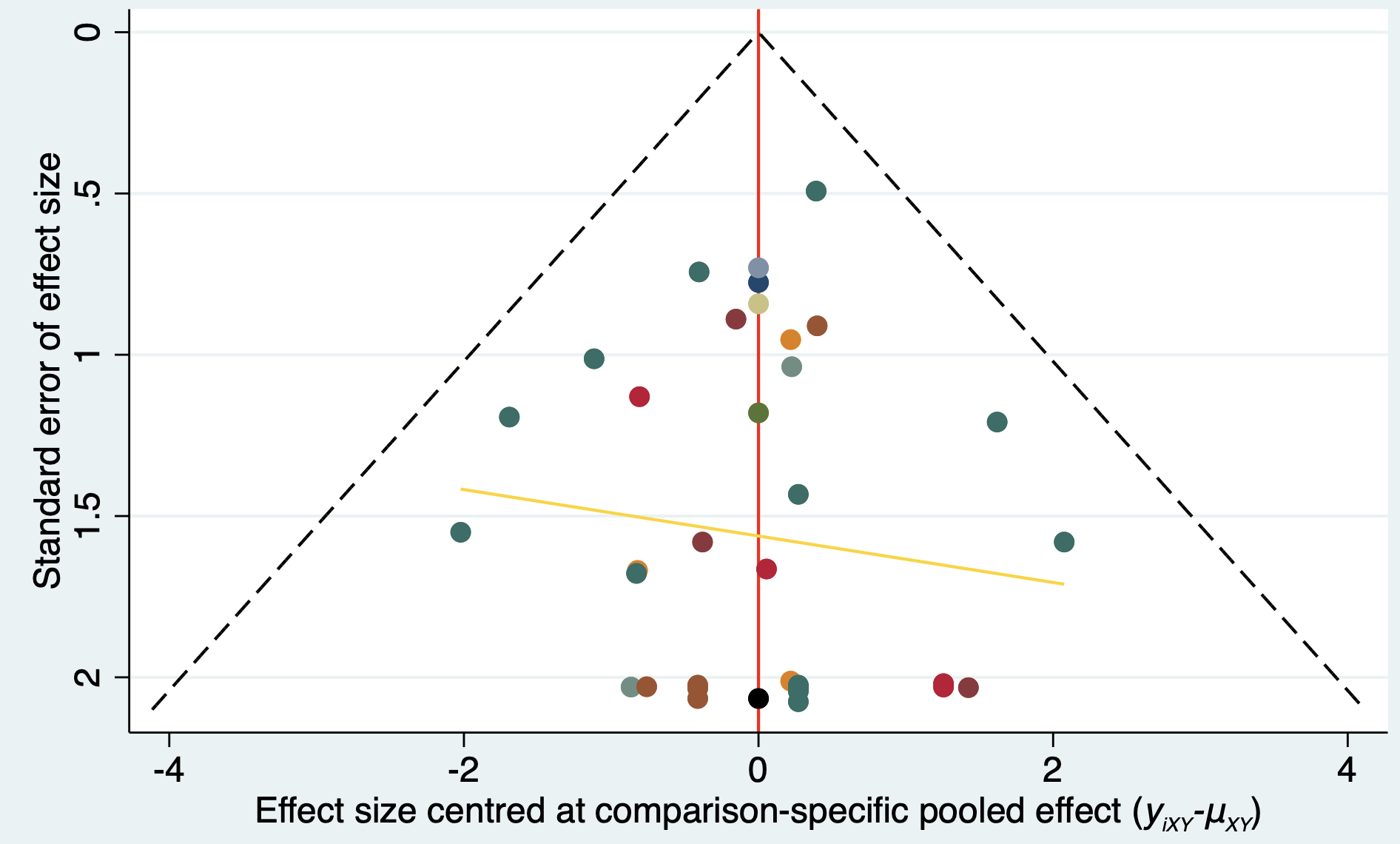


Fig. S12.4 Comparison-adjusted funnel plots for acceptability (Egger test: *p* = 0.386)

# Supplementary 13 League tables for specific NDDs

Table S13.1 League table for attention in children with ADHD

| MBE |  |  |  |  |  |
| --- | --- | --- | --- | --- | --- |
| 0.13 (-1.45,1.70) | MPA |  |  |  |  |
| 1.20 (-0.11,2.52) | 1.08 (-0.10,2.26) | NF |  |  |  |
| 1.32 (-0.74,3.37) | 1.19 (-0.87,3.25) | 0.12 (-1.71,1.94) | Exergaming |  |  |
| 1.44 (-0.01,2.89) | 1.31 (-0.15,2.77) | 0.24 (-0.86,1.33) | 0.12 (-1.33,1.58) | AE |  |
| **2.00 (0.89,3.10)** | **1.87 (0.61,3.14)** | 0.80 (-0.26,1.85) | 0.68 (-1.14,2.50) | 0.56 (-0.53,1.65) | UC |

*AE* aerobic exercise, *MBE* mind-body exercise, *MPA* multicomponent physical activity, *NF* neurofeedback, *UC* usual care. Results are presented as standardized mean differences (SMD) and 95% confidence intervals. SMD values more than 0.00 favor the column-defining treatment node for the NMA results. Estimates in bold denote significance at *p*<0.05.

Table S13.4 League table for memory in children with ADHD

| MPA |  |  |  |  |  |
| --- | --- | --- | --- | --- | --- |
| 0.38 (-0.35,1.11) | MBE |  |  |  |  |
| 0.63 (-0.22,1.48) | 0.25 (-0.56,1.06) | Exergaming |  |  |  |
| 0.66 (-0.11,1.42) | 0.28 (-0.52,1.07) | 0.03 (-0.94,0.99) | NF |  |  |
| **0.74 (0.14,1.35)** | 0.36 (-0.31,1.04) | 0.11 (-0.71,0.93) | 0.09 (-0.49,0.66) | AE |  |
| **0.81 (0.24,1.38)** | 0.43 (-0.07,0.93) | 0.18 (-0.45,0.82) | 0.16 (-0.57,0.88) | 0.07 (-0.45,0.59) | UC |

*AE* aerobic exercise, *MBE* mind-body exercise, *MPA* multicomponent physical activity, *NF* neurofeedback, *UC* usual care. Results are presented as standardized mean differences (SMD) and 95% confidence intervals. SMD values more than 0.00 favor the column-defining treatment node for the NMA results. Estimates in bold denote significance at *p*<0.05.

Table S13.3 League table for executive functions in children with ADHD

| MBE |  |  |  |  |  |
| --- | --- | --- | --- | --- | --- |
| **1.08 (0.12,2.05)** | MPA |  |  |  |  |
| **1.26 (0.24,2.27)** | 0.17 (-0.36,0.71) | Exergaming |  |  |  |
| **1.80 (0.56,3.04)** | 0.72 (-0.16,1.59) | 0.54 (-0.36,1.44) | NF |  |  |
| **1.82 (0.93,2.71)** | **0.74 (0.36,1.12)** | **0.57 (0.08,1.06)** | 0.02 (-0.84,0.89) | UC |  |
| **1.91 (0.87,2.95)** | **0.83 (0.26,1.40)** | **0.66 (0.06,1.25)** | 0.11 (-0.56,0.78) | 0.09 (-0.45,0.63) | AE |

*AE* aerobic exercise, *MBE* mind-body exercise, *MPA* multicomponent physical activity, *NF* neurofeedback, *UC* usual care. Results are presented as standardized mean differences (SMD) and 95% confidence intervals. SMD values more than 0.00 favor the column-defining treatment node for the NMA results. Estimates in bold denote significance at *p*<0.05.

Table S13.4 League table for executive functions in children with ASD

| MPA |  |  |  |  |  |
| --- | --- | --- | --- | --- | --- |
| -0.04 (-1.10,1.02) | Exergaming |  |  |  |  |
| 0.20 (-0.80,1.21) | 0.25 (-1.13,1.62) | MBE |  |  |  |
| 0.48 (-0.30,1.26) | 0.52 (-0.77,1.81) | 0.28 (-0.94,1.49) | AE |  |  |
| 0.74 (-0.81,2.29) | 0.78 (-1.03,2.59) | 0.53 (-0.65,1.71) | 0.26 (-1.44,1.95) | RT |  |
| **0.67 (0.18,1.17)** | 0.71 (-0.35,1.78) | 0.47 (-0.41,1.34) | 0.19 (-0.65,1.04) | -0.06 (-1.53,1.41) | UC |

*AE* aerobic exercise, *MBE* mind-body exercise, *MPA* multicomponent physical activity, *RT* relaxation techniques, *UC* usual care. Results are presented as standardized mean differences (SMD) and 95% confidence intervals. SMD values more than 0.00 favor the column-defining treatment node for the NMA results. Estimates in bold denote significance at *p*<0.05.

# Supplementary 14 Sensitivity Analyses

Given that no studies evaluating memory were classified as having a high risk of bias, sensitivity analysis was not performed for memory.

Table S14.1 League tables for attention after removing studies with high risk of bias

| MBE |  |  |  |  |  |
| --- | --- | --- | --- | --- | --- |
| 0.35 (-1.73,2.42) | Exergaming |  |  |  |  |
| 0.79 (-1.29,2.87) | 0.44 (-1.72,2.60) | MPA |  |  |  |
| 1.10 (-0.76,2.97) | 0.76 (-0.81,2.32) | 0.32 (-1.70,2.33) | AE |  |  |
| 1.13 (-0.66,2.92) | 0.78 (-1.20,2.76) | 0.34 (-1.81,2.49) | 0.02 (-1.51,1.56) | NF |  |
| **1.99 (0.54,3.45)** | **1.64 (0.08,3.21)** | 1.20 (-0.27,2.68) | 0.89 (-0.49,2.26) | 0.87 (-0.69,2.42) | UC |

*AE* aerobic exercise, *MBE* mind-body exercise, *MPA* multicomponent physical activity, *RT* relaxation techniques, *NF* neurofeedback, *UC* usual care. Results are presented as standardized mean differences (SMD) and 95% confidence intervals. SMD values more than 0.00 favor the column-defining treatment node. Estimates in bold denote significance at *p*<0.05

Table S14.2 League tables for executive functions after removing studies with high risk of bias

| Exergaming |  |  |  |  |  |  |
| --- | --- | --- | --- | --- | --- | --- |
| 0.10 (-0.95,1.15) | MBE |  |  |  |  |  |
| 0.13 (-0.45,0.71) | 0.03 (-0.93,0.99) | MPA |  |  |  |  |
| 0.63 (-0.98,2.25) | 0.53 (-0.69,1.76) | 0.50 (-1.05,2.06) | RT |  |  |  |
| 0.67 (-0.68,2.01) | 0.57 (-1.00,2.13) | 0.54 (-0.74,1.82) | 0.03 (-1.96,2.02) | NF |  |  |
| **0.78 (0.08,1.48)** | 0.68 (-0.39,1.75) | **0.65 (0.09,1.22)** | 0.15 (-1.48,1.77) | 0.11 (-1.03,1.26) | AE |  |
| **0.95 (0.40,1.49)** | 0.85 (-0.05,1.74) | **0.82 (0.48,1.16)** | 0.32 (-1.20,1.83) | 0.28 (-1.01,1.57) | 0.17 (-0.41,0.75) | UC |

*AE* aerobic exercise, *MBE* mind-body exercise, *MPA* multicomponent physical activity, *RT* relaxation techniques, *NF* neurofeedback, *UC* usual care. Results are presented as standardized mean differences (SMD) and 95% confidence intervals. SMD values more than 0.00 favor the column-defining treatment node. Estimates in bold denote significance at *p*<0.05

Table S14.3. League tables for acceptability after removing studies with high risk of bias

| MBE |  |  |  |  |  |  |
| --- | --- | --- | --- | --- | --- | --- |
| 0.81 (0.08,8.47) | NF |  |  |  |  |  |
| 0.63 (0.16,2.53) | 0.78 (0.10,6.11) | UC |  |  |  |  |
| 0.66 (0.13,3.45) | 0.82 (0.05,14.50) | 1.05 (0.12,9.13) | RT |  |  |  |
| 0.39 (0.08,1.81) | 0.48 (0.06,3.91) | 0.62 (0.31,1.22) | 0.59 (0.06,5.60) | MPA |  |  |
| 0.32 (0.05,1.85) | 0.39 (0.04,3.53) | 0.51 (0.17,1.52) | 0.48 (0.04,5.36) | 0.82 (0.27,2.51) | Exergaming |  |
| 0.23 (0.04,1.21) | 0.28 (0.04,2.01) | 0.36 (0.14,0.96) | 0.34 (0.03,3.59) | 0.59 (0.22,1.58) | 0.71 (0.23,2.19) | AE |

*AE* aerobic exercise, *MBE* mind-body exercise, *MPA* multicomponent physical activity, *RT* relaxation techniques, *NF* neurofeedback, *UC* usual care. Results are presented as standardized mean differences (SMD) and 95% confidence intervals. SMD values more than 0.00 favor the column-defining treatment node. Estimates in bold denote significance at *p*<0.05

# Supplementary 15 Meta-regression and subgroup analyses

Because undertaking subgroup analysis requires a minimum of ten studies [4], we restricted this analysis to memory and executive functions.

Table S15.1a Meta-regression analysis for attention

|  | ***k*** | **Coef. (SE)** | **95% CI** | **I^2^ (**%) | ***p*** |
| --- | --- | --- | --- | --- | --- |
| Sample size | 7 | -0.014 (0.019) | (-0.050, 0.023) | 90.44 | 0.463 |
| Total length (week) | 7 | 0.028 (0.015) | (-0.002, 0.058) | 87.47 | 0.064 |
| Total dose (min) | 7 | 0.000 (0.000) | (-0.00, 0.001) | 89.01 | 0.123 |

*k*=number of data points, coef.=regression coefficient, SE=standard error.

Table S15.1b Meta-regression analysis for memory

|  | ***k*** | **Coef. (SE)** | **95% CI** | **I^2^ (**%) | ***p*** |
| --- | --- | --- | --- | --- | --- |
| Sample size | 10 | 0.008 (0.019) | (-0.029, 0.045) | 73.10 | 0.665 |
| Total length (week) | 10 | -0.003 (0.060) | (-0.121, 0.114) | 73.72 | 0.959 |
| Total dose (min) | 10 | -0.000 (0.000) | (-0.001, 0.000) | 73.39 | 0.787 |

*k*=number of data points, coef.=regression coefficient, SE=standard error.

Table S15.1c Meta-regression analysis for excutive functions

|  | ***k*** | **Coef. (SE)** | **95% CI** | **I^2^ (**%) | ***p*** |
| --- | --- | --- | --- | --- | --- |
| Sample size | 24 | -0.006 (0.136) | (-0.324, 0.021) | 79.90 | 0.677 |
| Total length (week) | 24 | 0.003 (0.012) | (-0.020, 0.026) | 80.62 | 0.805 |
| Total dose (min) | 24 | 0.000 (0.000) | (-0.000, 0.000) | 80.45 | 0.562 |

*k*=number of data points, coef.=regression coefficient, SE=standard error.

Table S15.2a Subgroup analyses for memory

|  | ***k*** | **Hedges’ *g*** | **95% CI** | **I^2^ (**%) | **Test of heterogeneity** | | |
| --- | --- | --- | --- | --- | --- | --- | --- |
|  |  |  |  |  | **Q** | **df** | ***p*** |
| **NDDs category** |  |  |  |  | 24.16 | 3 | 0.000 |
| ADHD | 5 | 0.358 | (0.047, 0.668) | 15.45 |  |  |  |
| ASD | 3 | -0.060 | (-0.522, 0.402) | 25.49 |  |  |  |
| DCD | 1 | 1.810 | (1.159, 2.461) | . |  |  |  |
| SLD | 1 | 1.057 | (0.311, 1.803) | . |  |  |  |
| **Duration** |  |  |  |  | 0.11 | 1 | 0.735 |
| ≤ 50min | 4 | 0.571 | (-0.349, 1.490) | 86.02 |  |  |  |
| > 50min | 6 | 0.401 | (0.058, 0.745) | 38.52 |  |  |  |
| **Frequency** |  |  |  |  | 4.94 | 2 | 0.084 |
| < 3 session/week | 2 | 0.051 | (-0.711, 0.812) | 72.25 |  |  |  |
| 3 sessions/week | 5 | 0.860 | (0.296, 1.424) | 69.16 |  |  |  |
| > 3 sessions/week | 3 | 0.116 | (-0.296, 0.527) | 1.68 |  |  |  |
| **Length** |  |  |  |  | 1.84 | 1 | 0.175 |
| ≤ 8 weeks | 6 | 0.676 | (0.115, 1.237) | 72.08 |  |  |  |
| > 8 weeks | 4 | 0.184 | (-0.251, 0.620) | 52.52 |  |  |  |
| **Intensity** |  |  |  |  | 0.05 | 2 | 0.973 |
| LMPA | 2 | 0.145 | (-0.384, 0.675) | 3.65 |  |  |  |
| Moderate | 3 | 0.216 | (-0.355, 0.787) | 65.30 |  |  |  |
| MVPA | 2 | 0.133 | (-0.303, 0.569) | 0.21 |  |  |  |

*ADHD* attention-deficit/hyperactivity disorder, *ASD* autism spectrum disorder, *DCD* developmental coordination disorder, *LD* learning disorder, *LMPA* light-to-moderate physical activity; *MVPA* moderate-to-vigorous physical activity.

Table S15.2b. Subgroup analyses for executive functions

|  | ***k*** | **Hedges’ *g*** | **95% CI** | % | **Test of heterogeneity** | | |
| --- | --- | --- | --- | --- | --- | --- | --- |
|  |  |  |  |  | **Q** | **df** | ***p*** |
| **NDDs category** |  |  |  |  | 2.19 | 3 | 0.535 |
| ADHD | 13 | 0.779 | (0.444, 1.114) | 67.69 |  |  |  |
| ASD | 8 | 0.566 | (0.055, 1.077) | 72.84 |  |  |  |
| DCD | 2 | 2.391 | (-0.244, 5.025) | 67.69 |  |  |  |
| LD | 1 | 0.567 | (-0.144, 1.278) | . |  |  |  |
| **Duration** |  |  |  |  | 2.64 | 1 | 0.104 |
| ≤ 50min | 10 | 0.527 | (0.246, 0.809) | 37.65 |  |  |  |
| > 50min | 14 | 1.023 | (0.496, 1.551) | 85.79 |  |  |  |
| **Frequency** |  |  |  |  | 6.96 | 2 | 0.031 |
| < 3 session/week | 8 | 0.827 | (0.416, 1.238) | 61.53 |  |  |  |
| 3 sessions/week | 14 | 0.905 | (0.394, 1.416) | 85.24 |  |  |  |
| > 3 sessions/week | 2 | 0.071 | (-0.420, 0.563) | 0.00 |  |  |  |
| **Length** |  |  |  |  | 0.95 | 1 | 0.330 |
| ≤ 8 weeks | 12 | 0.641 | (0.111, 1.171) | 85.58 |  |  |  |
| > 8 weeks | 12 | 0.975 | (0.562, 1.338) | 71.82 |  |  |  |
| **Intensity** |  |  |  |  | 6.18 | 2 | 0.046 |
| LMPA | 4 | 0.057 | (-0.311, 0.425) | 0.40 |  |  |  |
| Moderate | 3 | 1.163 | (0.218, 2.108) | 80.72 |  |  |  |
| MVPA | 5 | 0.674 | (0.031, 1.316) | 79.00 |  |  |  |

*ADHD* attention-deficit/hyperactivity disorder, *ASD* autism spectrum disorder, *DCD* developmental coordination disorder, *LD* learning disorder, *LMPA* light-to-moderate physical activity; *MVPA* moderate-to-vigorous physical activity.

# Supplementary 16 Grading the evidence of the network meta-analysis using CINeMA

We used the CINeMA (Confidence in Network Meta-Analysis) framework, implemented in the web application CINeMA (https://cinema.ispm.unibe.ch/), to assess the certainty of evidence for cognitive functions [5,6]. The CINeMA framework consists of six domains that affect the level of evidence in the estimates of NMA: within‐study bias, reporting bias, indirectness, imprecision, heterogeneity, and incoherence.

To evaluate each network estimate, we used the following criteria:

1. Within-study bias: We assessed overall risk of bias using the Cochrane risk of bias tool 2. Within-study bias for each pairwise comparison was classified as “no concerns”, “some concerns”, or “major concerns” based on the weighted average overall risk of bias according to the contribution matrix.
2. Reporting bias: We inspected publication bias by comparison-adjusted funnel plots and Egger test results. We cannot completely rule out the possibility that some studies are still missing, and we carefully assumed some comparisons as “some concerns” according to funnel plot.
3. Indirectness: We evaluated whether populations, interventions, outcomes, and study settings represented the specific research questions. No clear evidence of violations of the transitivity assumption was found in this network, and meta-regression showed that potential modifiers did not significantly impact the primary result. Thus, indirectness was not downgraded.
4. Imprecision: We set a cutoff threshold of SMD 0.5 for clinical effectiveness [7] and assessed precision using treatment effects included in the 95% confidence interval relative to potentially clinically important differences for each pairwise comparison.
5. Heterogeneity: We considered heterogeneity based on the relationship between the 95% confidence intervals and the 95% prediction intervals with the clinically important differences defined above in the domain of imprecision.
6. Incoherence: We used the design-by-treatment model and did not detect any global inconsistency (*p*>0.05). We used side-splitting approach and did not detect any local inconsistency (*p*>0.05). Thus, incoherence was not downgraded.

We classified the overall judgment on the confidence in the NMA estimate for each comparison as “very low,” “low,” “moderate,” and “high.” The starting point for certainty was high, and it could be downgraded to one level for a rating of “some concerns” and two levels for a rating of “major concerns.” It is recommended to consider judgments on different domains jointly rather than in isolation, as imprecision, incoherence, and heterogeneity are interconnected.

In this NMA, the evidence was downgraded primarily due to within-study bias, imprecision, and heterogeneity concerns. Within-study bias was attributed to factors such as open-label studies, unblinded assessors, and improper handling of missing data, resulting in a rating of “some concern”. After excluding studies with high risk of bias assessed by the risk of bias tool 2, the results remained consistent, indicating the stability of the outcomes. Regarding imprecision, comparisons rated with “some concerns” did not contribute significantly to the findings in the league tables. Moderate heterogeneity, as observed in this study, might result in somewhat different conclusions but had a lesser impact on decision-making [5].

Table S16.1 CINeMA assessment for attention

| **Comparison** | **N of**  **studies** | **Within-study bias** | **Reporting bias** | **Indirectness** | **Imprecision** | **Heterogeneity** | **Incoherence** | **Confidence rating** |
| --- | --- | --- | --- | --- | --- | --- | --- | --- |
| AE:Exergaming | 1 | Some concerns | Low risk | No concerns | Some concerns | No concerns | No concerns | Low |
| AE:NF | 1 | Some concerns | Low risk | No concerns | Some concerns | No concerns | No concerns | Low |
| AE:UC | 1 | Some concerns | Low risk | No concerns | No concerns | Some concerns | No concerns | Low |
| Exergaming:UC | 1 | Some concerns | Low risk | No concerns | No concerns | Major concerns | No concerns | Very low |
| MBE:NF | 1 | Some concerns | Low risk | No concerns | No concerns | Some concerns | No concerns | Low |
| MBE:UC | 2 | Some concerns | Some concerns | No concerns | No concerns | Major concerns | No concerns | Very low |
| MPA:NF | 1 | Major concerns | Low risk | No concerns | Some concerns | No concerns | No concerns | Very low |
| MPA:UC | 3 | Major concerns | Some concerns | No concerns | No concerns | Major concerns | No concerns | Very low |
| NF:UC | 1 | Some concerns | Low risk | No concerns | Some concerns | No concerns | No concerns | Low |
| AE:MBE | 0 | Some concerns | Low risk | No concerns | No concerns | Some concerns | No concerns | Low |
| AE:MPA | 0 | Some concerns | Low risk | No concerns | Some concerns | No concerns | No concerns | Low |
| Exergaming:MBE | 0 | Some concerns | Low risk | No concerns | Some concerns | No concerns | No concerns | Low |
| Exergaming:MPA | 0 | Some concerns | Low risk | No concerns | Some concerns | No concerns | No concerns | Low |
| Exergaming:NF | 0 | Some concerns | Low risk | No concerns | Some concerns | No concerns | No concerns | Low |
| MBE:MPA | 0 | Some concerns | Low risk | No concerns | Some concerns | No concerns | No concerns | Low |

Table S16.2 CINeMA assessment for memory

| **Comparison** | **N of**  **studies** | **Within-study bias** | **Reporting bias** | **Indirectness** | **Imprecision** | **Heterogeneity** | **Incoherence** | **Confidence rating** |
| --- | --- | --- | --- | --- | --- | --- | --- | --- |
| AE:MPA | 2 | Some concerns | Low risk | No concerns | No concerns | Some concerns | No concerns | Low |
| AE:NF | 1 | Some concerns | Low risk | No concerns | Some concerns | No concerns | No concerns | Low |
| AE:UC | 2 | Some concerns | Low risk | No concerns | Some concerns | No concerns | No concerns | Low |
| Exergaming:UC | 2 | Some concerns | Low risk | No concerns | No concerns | Major concerns | No concerns | Very low |
| MBE:NF | 1 | Some concerns | Low risk | No concerns | Some concerns | No concerns | No concerns | Low |
| MBE:UC | 2 | Some concerns | Low risk | No concerns | No concerns | Some concerns | No concerns | Low |
| MPA:UC | 4 | Some concerns | Low risk | No concerns | No concerns | Some concerns | No concerns | Low |
| NF:UC | 1 | Some concerns | Low risk | No concerns | Some concerns | No concerns | No concerns | Low |
| AE:Exergaming | 0 | Some concerns | Low risk | No concerns | No concerns | Some concerns | No concerns | Low |
| AE:MBE | 0 | Some concerns | Low risk | No concerns | Some concerns | No concerns | No concerns | Low |
| Exergaming:MBE | 0 | Some concerns | Low risk | No concerns | Some concerns | No concerns | No concerns | Low |
| Exergaming:MPA | 0 | Some concerns | Low risk | No concerns | Some concerns | No concerns | No concerns | Low |
| Exergaming:NF | 0 | Some concerns | Low risk | No concerns | Some concerns | No concerns | No concerns | Low |
| MBE:MPA | 0 | Some concerns | Low risk | No concerns | Some concerns | No concerns | No concerns | Low |
| MPA:NF | 0 | Some concerns | Low risk | No concerns | Some concerns | No concerns | No concerns | Low |

Table S16.3 CINeMA assessment for executive functions

| **Comparison** | **N of**  **studies** | **Within-study bias** | **Reporting bias** | **Indirectness** | **Imprecision** | **Heterogeneity** | **Incoherence** | **Confidence rating** |
| --- | --- | --- | --- | --- | --- | --- | --- | --- |
| AE:Exergaming | 1 | Some concerns | Low risk | No concerns | No concerns | Some concerns | No concerns | Low |
| AE:MPA | 3 | Some concerns | Low risk | No concerns | No concerns | Major concerns | No concerns | Very low |
| AE:NF | 1 | Some concerns | Low risk | No concerns | Some concerns | No concerns | No concerns | Low |
| AE:UC | 2 | Some concerns | Low risk | No concerns | No concerns | Some concerns | No concerns | Low |
| Exergaming:MPA | 2 | Some concerns | Low risk | No concerns | No concerns | Some concerns | No concerns | Low |
| Exergaming:UC | 4 | Some concerns | Some concerns | No concerns | No concerns | Some concerns | No concerns | Very low |
| MBE:RT | 1 | Some concerns | Low risk | No concerns | Some concerns | No concerns | No concerns | Low |
| MBE:UC | 3 | Some concerns | High risk | No concerns | No concerns | Some concerns | No concerns | Very low |
| MPA:UC | 15 | Some concerns | Low risk | No concerns | No concerns | Some concerns | No concerns | Low |
| AE:MBE | 0 | Some concerns | Low risk | No concerns | No concerns | Some concerns | No concerns | Low |
| AE:RT | 0 | Some concerns | Low risk | No concerns | Some concerns | No concerns | No concerns | Low |
| Exergaming:MBE | 0 | Some concerns | Low risk | No concerns | Some concerns | No concerns | No concerns | Low |
| Exergaming:NF | 0 | Some concerns | Low risk | No concerns | Some concerns | No concerns | No concerns | Low |
| Exergaming:RT | 0 | Some concerns | Low risk | No concerns | Some concerns | No concerns | No concerns | Low |
| MBE:MPA | 0 | Some concerns | Low risk | No concerns | Some concerns | No concerns | No concerns | Low |
| MBE:NF | 0 | Some concerns | Low risk | No concerns | Some concerns | No concerns | No concerns | Low |
| MPA:NF | 0 | Some concerns | Low risk | No concerns | Some concerns | No concerns | No concerns | Low |
| MPA:RT | 0 | Some concerns | Low risk | No concerns | Some concerns | No concerns | No concerns | Low |
| NF:RT | 0 | Some concerns | Low risk | No concerns | Some concerns | No concerns | No concerns | Low |
| NF:UC | 0 | Some concerns | Low risk | No concerns | Some concerns | No concerns | No concerns | Low |
| RT:UC | 0 | Some concerns | Low risk | No concerns | Some concerns | No concerns | No concerns | Low |

# Reference

1. Rhodes KM, Turner RM, Higgins JPT. Predictive distributions were developed for the extent of heterogeneity in meta-analyses of continuous outcome data. J Clin Epidemiol 2015; 68(1): 52-60. https://doi.org/10.1016/j.jclinepi.2014.08.012.
2. Turner RM, Davey J, Clarke MJ, Thompson SG, Higgins JP. Predicting the extent of heterogeneity in meta-analysis, using empirical data from the Cochrane Database of Systematic Reviews. Int J Epidemiol 2012; 41(3): 818-27. https://doi.org/10.1093/ije/dys041.
3. Ge L, Sadeghirad B, Ball GDC, et al. Comparison of dietary macronutrient patterns of 14 popular named dietary programmes for weight and cardiovascular risk factor reduction in adults: systematic review and network meta-analysis of randomised trials. BMJ 2020; 369: m696. https://doi.org/10.1136/bmj.m696.
4. Deeks JJ, Higgins JPT, Altman DG. Analysing data and undertaking meta-analyses. Cochrane Handbook for Systematic Reviews of Interventions; 2019: 241-84.
5. Nikolakopoulou A, Higgins JP, Papakonstantinou T, et al. CINeMA: an approach for assessing confidence in the results of a network meta-analysis. PLoS med 2020; 17(4): e1003082. https://doi.org/10.1371/journal.pmed.1003082.
6. Papakonstantinou T, Nikolakopoulou A, Higgins JP, Egger M, Salanti G. CINeMA: software for semiautomated assessment of the confidence in the results of network meta‐analysis. Campbell Syst Rev 2020; 16(1): e1080. https://doi.org/10.1002/cl2.1080.
7. Guyatt GH, Oxman AD, Kunz R, et al. GRADE guidelines 6. Rating the quality of evidence—imprecision. J Clin Epidemiol 2011; 64(12): 1283-93. https://doi.org/10.1016/j.jclinepi.2011.01.012.
